# Search for a light charged Higgs boson in the decay channel $H^{+} \rightarrow c\bar{s}$ in $t\bar{t}$ events using *pp* collisions at $\sqrt{s} = 7\ \mathrm{TeV}$ with the ATLAS detector

**DOI:** 10.1140/epjc/s10052-013-2465-z

**Published:** 2013-06-06

**Authors:** The ATLAS Collaboration, G. Aad, T. Abajyan, B. Abbott, J. Abdallah, S. Abdel Khalek, A. A. Abdelalim, O. Abdinov, R. Aben, B. Abi, M. Abolins, O. S. AbouZeid, H. Abramowicz, H. Abreu, B. S. Acharya, L. Adamczyk, D. L. Adams, T. N. Addy, J. Adelman, S. Adomeit, P. Adragna, T. Adye, S. Aefsky, J. A. Aguilar-Saavedra, M. Agustoni, S. P. Ahlen, F. Ahles, A. Ahmad, M. Ahsan, G. Aielli, T. P. A. Åkesson, G. Akimoto, A. V. Akimov, M. A. Alam, J. Albert, S. Albrand, M. Aleksa, I. N. Aleksandrov, F. Alessandria, C. Alexa, G. Alexander, G. Alexandre, T. Alexopoulos, M. Alhroob, M. Aliev, G. Alimonti, J. Alison, B. M. M. Allbrooke, L. J. Allison, P. P. Allport, S. E. Allwood-Spiers, J. Almond, A. Aloisio, R. Alon, A. Alonso, F. Alonso, A. Altheimer, B. Alvarez Gonzalez, M. G. Alviggi, K. Amako, C. Amelung, V. V. Ammosov, S. P. Amor Dos Santos, A. Amorim, S. Amoroso, N. Amram, C. Anastopoulos, L. S. Ancu, N. Andari, T. Andeen, C. F. Anders, G. Anders, K. J. Anderson, A. Andreazza, V. Andrei, M-L. Andrieux, X. S. Anduaga, S. Angelidakis, P. Anger, A. Angerami, F. Anghinolfi, A. Anisenkov, N. Anjos, A. Annovi, A. Antonaki, M. Antonelli, A. Antonov, J. Antos, F. Anulli, M. Aoki, S. Aoun, L. Aperio Bella, R. Apolle, G. Arabidze, I. Aracena, Y. Arai, A. T. H. Arce, S. Arfaoui, J-F. Arguin, S. Argyropoulos, E. Arik, M. Arik, A. J. Armbruster, O. Arnaez, V. Arnal, A. Artamonov, G. Artoni, D. Arutinov, S. Asai, S. Ask, B. Åsman, D. Asner, L. Asquith, K. Assamagan, A. Astbury, M. Atkinson, B. Aubert, E. Auge, K. Augsten, M. Aurousseau, G. Avolio, D. Axen, G. Azuelos, Y. Azuma, M. A. Baak, G. Baccaglioni, C. Bacci, A. M. Bach, H. Bachacou, K. Bachas, M. Backes, M. Backhaus, J. Backus Mayes, E. Badescu, P. Bagnaia, Y. Bai, D. C. Bailey, T. Bain, J. T. Baines, O. K. Baker, S. Baker, P. Balek, E. Banas, P. Banerjee, Sw. Banerjee, D. Banfi, A. Bangert, V. Bansal, H. S. Bansil, L. Barak, S. P. Baranov, T. Barber, E. L. Barberio, D. Barberis, M. Barbero, D. Y. Bardin, T. Barillari, M. Barisonzi, T. Barklow, N. Barlow, B. M. Barnett, R. M. Barnett, A. Baroncelli, G. Barone, A. J. Barr, F. Barreiro, J. Barreiro Guimarães da Costa, R. Bartoldus, A. E. Barton, V. Bartsch, A. Basye, R. L. Bates, L. Batkova, J. R. Batley, A. Battaglia, M. Battistin, F. Bauer, H. S. Bawa, S. Beale, T. Beau, P. H. Beauchemin, R. Beccherle, P. Bechtle, H. P. Beck, K. Becker, S. Becker, M. Beckingham, K. H. Becks, A. J. Beddall, A. Beddall, S. Bedikian, V. A. Bednyakov, C. P. Bee, L. J. Beemster, M. Begel, S. Behar Harpaz, P. K. Behera, M. Beimforde, C. Belanger-Champagne, P. J. Bell, W. H. Bell, G. Bella, L. Bellagamba, M. Bellomo, A. Belloni, O. Beloborodova, K. Belotskiy, O. Beltramello, O. Benary, D. Benchekroun, K. Bendtz, N. Benekos, Y. Benhammou, E. Benhar Noccioli, J. A. Benitez Garcia, D. P. Benjamin, M. Benoit, J. R. Bensinger, K. Benslama, S. Bentvelsen, D. Berge, E. Bergeaas Kuutmann, N. Berger, F. Berghaus, E. Berglund, J. Beringer, P. Bernat, R. Bernhard, C. Bernius, T. Berry, C. Bertella, A. Bertin, F. Bertolucci, M. I. Besana, G. J. Besjes, N. Besson, S. Bethke, W. Bhimji, R. M. Bianchi, L. Bianchini, M. Bianco, O. Biebel, S. P. Bieniek, K. Bierwagen, J. Biesiada, M. Biglietti, H. Bilokon, M. Bindi, S. Binet, A. Bingul, C. Bini, C. Biscarat, B. Bittner, C. W. Black, J. E. Black, K. M. Black, R. E. Blair, J.-B. Blanchard, T. Blazek, I. Bloch, C. Blocker, J. Blocki, W. Blum, U. Blumenschein, G. J. Bobbink, V. S. Bobrovnikov, S. S. Bocchetta, A. Bocci, C. R. Boddy, M. Boehler, J. Boek, T. T. Boek, N. Boelaert, J. A. Bogaerts, A. Bogdanchikov, A. Bogouch, C. Bohm, J. Bohm, V. Boisvert, T. Bold, V. Boldea, N. M. Bolnet, M. Bomben, M. Bona, M. Boonekamp, S. Bordoni, C. Borer, A. Borisov, G. Borissov, I. Borjanovic, M. Borri, S. Borroni, J. Bortfeldt, V. Bortolotto, K. Bos, D. Boscherini, M. Bosman, H. Boterenbrood, J. Bouchami, J. Boudreau, E. V. Bouhova-Thacker, D. Boumediene, C. Bourdarios, N. Bousson, A. Boveia, J. Boyd, I. R. Boyko, I. Bozovic-Jelisavcic, J. Bracinik, P. Branchini, A. Brandt, G. Brandt, O. Brandt, U. Bratzler, B. Brau, J. E. Brau, H. M. Braun, S. F. Brazzale, B. Brelier, J. Bremer, K. Brendlinger, R. Brenner, S. Bressler, T. M. Bristow, D. Britton, F. M. Brochu, I. Brock, R. Brock, F. Broggi, C. Bromberg, J. Bronner, G. Brooijmans, T. Brooks, W. K. Brooks, G. Brown, P. A. Bruckman de Renstrom, D. Bruncko, R. Bruneliere, S. Brunet, A. Bruni, G. Bruni, M. Bruschi, L. Bryngemark, T. Buanes, Q. Buat, F. Bucci, J. Buchanan, P. Buchholz, R. M. Buckingham, A. G. Buckley, S. I. Buda, I. A. Budagov, B. Budick, V. Büscher, L. Bugge, O. Bulekov, A. C. Bundock, M. Bunse, T. Buran, H. Burckhart, S. Burdin, T. Burgess, S. Burke, E. Busato, P. Bussey, C. P. Buszello, B. Butler, J. M. Butler, C. M. Buttar, J. M. Butterworth, W. Buttinger, M. Byszewski, S. Cabrera Urbán, D. Caforio, O. Cakir, P. Calafiura, G. Calderini, P. Calfayan, R. Calkins, L. P. Caloba, R. Caloi, D. Calvet, S. Calvet, R. Camacho Toro, P. Camarri, D. Cameron, L. M. Caminada, R. Caminal Armadans, S. Campana, M. Campanelli, V. Canale, F. Canelli, A. Canepa, J. Cantero, R. Cantrill, M. D. M. Capeans Garrido, I. Caprini, M. Caprini, D. Capriotti, M. Capua, R. Caputo, R. Cardarelli, T. Carli, G. Carlino, L. Carminati, S. Caron, E. Carquin, G. D. Carrillo-Montoya, A. A. Carter, J. R. Carter, J. Carvalho, D. Casadei, M. P. Casado, M. Cascella, C. Caso, A. M. Castaneda Hernandez, E. Castaneda-Miranda, V. Castillo Gimenez, N. F. Castro, G. Cataldi, P. Catastini, A. Catinaccio, J. R. Catmore, A. Cattai, G. Cattani, S. Caughron, V. Cavaliere, P. Cavalleri, D. Cavalli, M. Cavalli-Sforza, V. Cavasinni, F. Ceradini, A. S. Cerqueira, A. Cerri, L. Cerrito, F. Cerutti, S. A. Cetin, A. Chafaq, D. Chakraborty, I. Chalupkova, K. Chan, P. Chang, B. Chapleau, J. D. Chapman, J. W. Chapman, D. G. Charlton, V. Chavda, C. A. Chavez Barajas, S. Cheatham, S. Chekanov, S. V. Chekulaev, G. A. Chelkov, M. A. Chelstowska, C. Chen, H. Chen, S. Chen, X. Chen, Y. Chen, Y. Cheng, A. Cheplakov, R. Cherkaoui El Moursli, V. Chernyatin, E. Cheu, S. L. Cheung, L. Chevalier, G. Chiefari, L. Chikovani, J. T. Childers, A. Chilingarov, G. Chiodini, A. S. Chisholm, R. T. Chislett, A. Chitan, M. V. Chizhov, G. Choudalakis, S. Chouridou, I. A. Christidi, A. Christov, D. Chromek-Burckhart, M. L. Chu, J. Chudoba, G. Ciapetti, A. K. Ciftci, R. Ciftci, D. Cinca, V. Cindro, A. Ciocio, M. Cirilli, P. Cirkovic, Z. H. Citron, M. Citterio, M. Ciubancan, A. Clark, P. J. Clark, R. N. Clarke, W. Cleland, J. C. Clemens, B. Clement, C. Clement, Y. Coadou, M. Cobal, A. Coccaro, J. Cochran, L. Coffey, J. G. Cogan, J. Coggeshall, J. Colas, S. Cole, A. P. Colijn, N. J. Collins, C. Collins-Tooth, J. Collot, T. Colombo, G. Colon, G. Compostella, P. Conde Muiño, E. Coniavitis, M. C. Conidi, S. M. Consonni, V. Consorti, S. Constantinescu, C. Conta, G. Conti, F. Conventi, M. Cooke, B. D. Cooper, A. M. Cooper-Sarkar, K. Copic, T. Cornelissen, M. Corradi, F. Corriveau, A. Cortes-Gonzalez, G. Cortiana, G. Costa, M. J. Costa, D. Costanzo, D. Côté, G. Cottin, L. Courneyea, G. Cowan, B. E. Cox, K. Cranmer, F. Crescioli, M. Cristinziani, G. Crosetti, S. Crépé-Renaudin, C.-M. Cuciuc, C. Cuenca Almenar, T. Cuhadar Donszelmann, J. Cummings, M. Curatolo, C. J. Curtis, C. Cuthbert, P. Cwetanski, H. Czirr, P. Czodrowski, Z. Czyczula, S. D’Auria, M. D’Onofrio, A. D’Orazio, M. J. Da Cunha Sargedas De Sousa, C. Da Via, W. Dabrowski, A. Dafinca, T. Dai, F. Dallaire, C. Dallapiccola, M. Dam, M. Dameri, D. S. Damiani, H. O. Danielsson, V. Dao, G. Darbo, G. L. Darlea, J. A. Dassoulas, W. Davey, T. Davidek, N. Davidson, R. Davidson, E. Davies, M. Davies, O. Davignon, A. R. Davison, Y. Davygora, E. Dawe, I. Dawson, R. K. Daya-Ishmukhametova, K. De, R. de Asmundis, S. De Castro, S. De Cecco, J. de Graat, N. De Groot, P. de Jong, C. De La Taille, H. De la Torre, F. De Lorenzi, L. De Nooij, D. De Pedis, A. De Salvo, U. De Sanctis, A. De Santo, J. B. De Vivie De Regie, G. De Zorzi, W. J. Dearnaley, R. Debbe, C. Debenedetti, B. Dechenaux, D. V. Dedovich, J. Degenhardt, J. Del Peso, T. Del Prete, T. Delemontex, M. Deliyergiyev, A. Dell’Acqua, L. Dell’Asta, M. Della Pietra, D. della Volpe, M. Delmastro, P. A. Delsart, C. Deluca, S. Demers, M. Demichev, B. Demirkoz, S. P. Denisov, D. Derendarz, J. E. Derkaoui, F. Derue, P. Dervan, K. Desch, E. Devetak, P. O. Deviveiros, A. Dewhurst, B. DeWilde, S. Dhaliwal, R. Dhullipudi, A. Di Ciaccio, L. Di Ciaccio, C. Di Donato, A. Di Girolamo, B. Di Girolamo, S. Di Luise, A. Di Mattia, B. Di Micco, R. Di Nardo, A. Di Simone, R. Di Sipio, M. A. Diaz, E. B. Diehl, J. Dietrich, T. A. Dietzsch, S. Diglio, K. Dindar Yagci, J. Dingfelder, F. Dinut, C. Dionisi, P. Dita, S. Dita, F. Dittus, F. Djama, T. Djobava, M. A. B. do Vale, A. Do Valle Wemans, T. K. O. Doan, M. Dobbs, D. Dobos, E. Dobson, J. Dodd, C. Doglioni, T. Doherty, Y. Doi, J. Dolejsi, Z. Dolezal, B. A. Dolgoshein, T. Dohmae, M. Donadelli, J. Donini, J. Dopke, A. Doria, A. Dos Anjos, A. Dotti, M. T. Dova, A. D. Doxiadis, A. T. Doyle, N. Dressnandt, M. Dris, J. Dubbert, S. Dube, E. Dubreuil, E. Duchovni, G. Duckeck, D. Duda, A. Dudarev, F. Dudziak, M. Dührssen, I. P. Duerdoth, L. Duflot, M-A. Dufour, L. Duguid, M. Dunford, H. Duran Yildiz, R. Duxfield, M. Dwuznik, M. Düren, W. L. Ebenstein, J. Ebke, S. Eckweiler, W. Edson, C. A. Edwards, N. C. Edwards, W. Ehrenfeld, T. Eifert, G. Eigen, K. Einsweiler, E. Eisenhandler, T. Ekelof, M. El Kacimi, M. Ellert, S. Elles, F. Ellinghaus, K. Ellis, N. Ellis, J. Elmsheuser, M. Elsing, D. Emeliyanov, R. Engelmann, A. Engl, B. Epp, J. Erdmann, A. Ereditato, D. Eriksson, J. Ernst, M. Ernst, J. Ernwein, D. Errede, S. Errede, E. Ertel, M. Escalier, H. Esch, C. Escobar, X. Espinal Curull, B. Esposito, F. Etienne, A. I. Etienvre, E. Etzion, D. Evangelakou, H. Evans, L. Fabbri, C. Fabre, R. M. Fakhrutdinov, S. Falciano, Y. Fang, M. Fanti, A. Farbin, A. Farilla, J. Farley, T. Farooque, S. Farrell, S. M. Farrington, P. Farthouat, F. Fassi, P. Fassnacht, D. Fassouliotis, B. Fatholahzadeh, A. Favareto, L. Fayard, P. Federic, O. L. Fedin, W. Fedorko, M. Fehling-Kaschek, L. Feligioni, C. Feng, E. J. Feng, A. B. Fenyuk, J. Ferencei, W. Fernando, S. Ferrag, J. Ferrando, V. Ferrara, A. Ferrari, P. Ferrari, R. Ferrari, D. E. Ferreira de Lima, A. Ferrer, D. Ferrere, C. Ferretti, A. Ferretto Parodi, M. Fiascaris, F. Fiedler, A. Filipčič, F. Filthaut, M. Fincke-Keeler, M. C. N. Fiolhais, L. Fiorini, A. Firan, G. Fischer, M. J. Fisher, E. A. Fitzgerald, M. Flechl, I. Fleck, J. Fleckner, P. Fleischmann, S. Fleischmann, G. Fletcher, T. Flick, A. Floderus, L. R. Flores Castillo, A. C. Florez Bustos, M. J. Flowerdew, T. Fonseca Martin, A. Formica, A. Forti, D. Fortin, D. Fournier, A. J. Fowler, H. Fox, P. Francavilla, M. Franchini, S. Franchino, D. Francis, T. Frank, M. Franklin, S. Franz, M. Fraternali, S. Fratina, S. T. French, C. Friedrich, F. Friedrich, D. Froidevaux, J. A. Frost, C. Fukunaga, E. Fullana Torregrosa, B. G. Fulsom, J. Fuster, C. Gabaldon, O. Gabizon, S. Gadatsch, T. Gadfort, S. Gadomski, G. Gagliardi, P. Gagnon, C. Galea, B. Galhardo, E. J. Gallas, V. Gallo, B. J. Gallop, P. Gallus, K. K. Gan, Y. S. Gao, A. Gaponenko, F. Garberson, M. Garcia-Sciveres, C. García, J. E. García Navarro, R. W. Gardner, N. Garelli, V. Garonne, C. Gatti, G. Gaudio, B. Gaur, L. Gauthier, P. Gauzzi, I. L. Gavrilenko, C. Gay, G. Gaycken, E. N. Gazis, P. Ge, Z. Gecse, C. N. P. Gee, D. A. A. Geerts, Ch. Geich-Gimbel, K. Gellerstedt, C. Gemme, A. Gemmell, M. H. Genest, S. Gentile, M. George, S. George, D. Gerbaudo, P. Gerlach, A. Gershon, C. Geweniger, H. Ghazlane, N. Ghodbane, B. Giacobbe, S. Giagu, V. Giangiobbe, F. Gianotti, B. Gibbard, A. Gibson, S. M. Gibson, M. Gilchriese, D. Gillberg, A. R. Gillman, D. M. Gingrich, J. Ginzburg, N. Giokaris, M. P. Giordani, R. Giordano, F. M. Giorgi, P. Giovannini, P. F. Giraud, D. Giugni, M. Giunta, B. K. Gjelsten, L. K. Gladilin, C. Glasman, J. Glatzer, A. Glazov, G. L. Glonti, J. R. Goddard, J. Godfrey, J. Godlewski, M. Goebel, T. Göpfert, C. Goeringer, C. Gössling, S. Goldfarb, T. Golling, D. Golubkov, A. Gomes, L. S. Gomez Fajardo, R. Gonçalo, J. Goncalves Pinto Firmino Da Costa, L. Gonella, S. González de la Hoz, G. Gonzalez Parra, M. L. Gonzalez Silva, S. Gonzalez-Sevilla, J. J. Goodson, L. Goossens, P. A. Gorbounov, H. A. Gordon, I. Gorelov, G. Gorfine, B. Gorini, E. Gorini, A. Gorišek, E. Gornicki, A. T. Goshaw, M. Gosselink, M. I. Gostkin, I. Gough Eschrich, M. Gouighri, D. Goujdami, M. P. Goulette, A. G. Goussiou, C. Goy, S. Gozpinar, I. Grabowska-Bold, P. Grafström, K-J. Grahn, E. Gramstad, F. Grancagnolo, S. Grancagnolo, V. Grassi, V. Gratchev, H. M. Gray, J. A. Gray, E. Graziani, O. G. Grebenyuk, T. Greenshaw, Z. D. Greenwood, K. Gregersen, I. M. Gregor, P. Grenier, J. Griffiths, N. Grigalashvili, A. A. Grillo, K. Grimm, S. Grinstein, Ph. Gris, Y. V. Grishkevich, J.-F. Grivaz, A. Grohsjean, E. Gross, J. Grosse-Knetter, J. Groth-Jensen, K. Grybel, D. Guest, C. Guicheney, E. Guido, T. Guillemin, S. Guindon, U. Gul, J. Gunther, B. Guo, J. Guo, P. Gutierrez, N. Guttman, O. Gutzwiller, C. Guyot, C. Gwenlan, C. B. Gwilliam, A. Haas, S. Haas, C. Haber, H. K. Hadavand, D. R. Hadley, P. Haefner, F. Hahn, Z. Hajduk, H. Hakobyan, D. Hall, G. Halladjian, K. Hamacher, P. Hamal, K. Hamano, M. Hamer, A. Hamilton, S. Hamilton, L. Han, K. Hanagaki, K. Hanawa, M. Hance, C. Handel, P. Hanke, J. R. Hansen, J. B. Hansen, J. D. Hansen, P. H. Hansen, P. Hansson, K. Hara, T. Harenberg, S. Harkusha, D. Harper, R. D. Harrington, O. M. Harris, J. Hartert, F. Hartjes, T. Haruyama, A. Harvey, S. Hasegawa, Y. Hasegawa, S. Hassani, S. Haug, M. Hauschild, R. Hauser, M. Havranek, C. M. Hawkes, R. J. Hawkings, A. D. Hawkins, T. Hayakawa, T. Hayashi, D. Hayden, C. P. Hays, H. S. Hayward, S. J. Haywood, S. J. Head, V. Hedberg, L. Heelan, S. Heim, B. Heinemann, S. Heisterkamp, L. Helary, C. Heller, M. Heller, S. Hellman, D. Hellmich, C. Helsens, R. C. W. Henderson, M. Henke, A. Henrichs, A. M. Henriques Correia, S. Henrot-Versille, C. Hensel, C. M. Hernandez, Y. Hernández Jiménez, R. Herrberg, G. Herten, R. Hertenberger, L. Hervas, G. G. Hesketh, N. P. Hessey, R. Hickling, E. Higón-Rodriguez, J. C. Hill, K. H. Hiller, S. Hillert, S. J. Hillier, I. Hinchliffe, E. Hines, M. Hirose, F. Hirsch, D. Hirschbuehl, J. Hobbs, N. Hod, M. C. Hodgkinson, P. Hodgson, A. Hoecker, M. R. Hoeferkamp, J. Hoffman, D. Hoffmann, M. Hohlfeld, M. Holder, S. O. Holmgren, T. Holy, J. L. Holzbauer, T. M. Hong, L. Hooft van Huysduynen, S. Horner, J-Y. Hostachy, S. Hou, A. Hoummada, J. Howard, J. Howarth, I. Hristova, J. Hrivnac, T. Hryn’ova, P. J. Hsu, S.-C. Hsu, D. Hu, Z. Hubacek, F. Hubaut, F. Huegging, A. Huettmann, T. B. Huffman, E. W. Hughes, G. Hughes, M. Huhtinen, M. Hurwitz, N. Huseynov, J. Huston, J. Huth, G. Iacobucci, G. Iakovidis, M. Ibbotson, I. Ibragimov, L. Iconomidou-Fayard, J. Idarraga, P. Iengo, O. Igonkina, Y. Ikegami, M. Ikeno, D. Iliadis, N. Ilic, T. Ince, P. Ioannou, M. Iodice, K. Iordanidou, V. Ippolito, A. Irles Quiles, C. Isaksson, M. Ishino, M. Ishitsuka, R. Ishmukhametov, C. Issever, S. Istin, A. V. Ivashin, W. Iwanski, H. Iwasaki, J. M. Izen, V. Izzo, B. Jackson, J. N. Jackson, P. Jackson, M. R. Jaekel, V. Jain, K. Jakobs, S. Jakobsen, T. Jakoubek, J. Jakubek, D. O. Jamin, D. K. Jana, E. Jansen, H. Jansen, J. Janssen, A. Jantsch, M. Janus, R. C. Jared, G. Jarlskog, L. Jeanty, I. Jen-La Plante, G.-Y. Jeng, D. Jennens, P. Jenni, A. E. Loevschall-Jensen, P. Jež, S. Jézéquel, M. K. Jha, H. Ji, W. Ji, J. Jia, Y. Jiang, M. Jimenez Belenguer, S. Jin, O. Jinnouchi, M. D. Joergensen, D. Joffe, M. Johansen, K. E. Johansson, P. Johansson, S. Johnert, K. A. Johns, K. Jon-And, G. Jones, R. W. L. Jones, T. J. Jones, C. Joram, P. M. Jorge, K. D. Joshi, J. Jovicevic, T. Jovin, X. Ju, C. A. Jung, R. M. Jungst, V. Juranek, P. Jussel, A. Juste Rozas, S. Kabana, M. Kaci, A. Kaczmarska, P. Kadlecik, M. Kado, H. Kagan, M. Kagan, E. Kajomovitz, S. Kalinin, L. V. Kalinovskaya, S. Kama, N. Kanaya, M. Kaneda, S. Kaneti, T. Kanno, V. A. Kantserov, J. Kanzaki, B. Kaplan, A. Kapliy, D. Kar, M. Karagounis, K. Karakostas, M. Karnevskiy, V. Kartvelishvili, A. N. Karyukhin, L. Kashif, G. Kasieczka, R. D. Kass, A. Kastanas, Y. Kataoka, J. Katzy, V. Kaushik, K. Kawagoe, T. Kawamoto, G. Kawamura, S. Kazama, V. F. Kazanin, M. Y. Kazarinov, R. Keeler, P. T. Keener, R. Kehoe, M. Keil, G. D. Kekelidze, J. S. Keller, M. Kenyon, H. Keoshkerian, O. Kepka, N. Kerschen, B. P. Kerševan, S. Kersten, K. Kessoku, J. Keung, F. Khalil-zada, H. Khandanyan, A. Khanov, D. Kharchenko, A. Khodinov, A. Khomich, T. J. Khoo, G. Khoriauli, A. Khoroshilov, V. Khovanskiy, E. Khramov, J. Khubua, H. Kim, S. H. Kim, N. Kimura, O. Kind, B. T. King, M. King, R. S. B. King, J. Kirk, A. E. Kiryunin, T. Kishimoto, D. Kisielewska, T. Kitamura, T. Kittelmann, K. Kiuchi, E. Kladiva, M. Klein, U. Klein, K. Kleinknecht, M. Klemetti, A. Klier, P. Klimek, A. Klimentov, R. Klingenberg, J. A. Klinger, E. B. Klinkby, T. Klioutchnikova, P. F. Klok, S. Klous, E.-E. Kluge, T. Kluge, P. Kluit, S. Kluth, E. Kneringer, E. B. F. G. Knoops, A. Knue, B. R. Ko, T. Kobayashi, M. Kobel, M. Kocian, P. Kodys, K. Köneke, A. C. König, S. Koenig, L. Köpke, F. Koetsveld, P. Koevesarki, T. Koffas, E. Koffeman, L. A. Kogan, S. Kohlmann, F. Kohn, Z. Kohout, T. Kohriki, T. Koi, G. M. Kolachev, H. Kolanoski, V. Kolesnikov, I. Koletsou, J. Koll, A. A. Komar, Y. Komori, T. Kondo, T. Kono, A. I. Kononov, R. Konoplich, N. Konstantinidis, R. Kopeliansky, S. Koperny, K. Korcyl, K. Kordas, A. Korn, A. Korol, I. Korolkov, E. V. Korolkova, V. A. Korotkov, O. Kortner, S. Kortner, V. V. Kostyukhin, S. Kotov, V. M. Kotov, A. Kotwal, C. Kourkoumelis, V. Kouskoura, A. Koutsman, R. Kowalewski, T. Z. Kowalski, W. Kozanecki, A. S. Kozhin, V. Kral, V. A. Kramarenko, G. Kramberger, M. W. Krasny, A. Krasznahorkay, J. K. Kraus, A. Kravchenko, S. Kreiss, F. Krejci, J. Kretzschmar, K. Kreutzfeldt, N. Krieger, P. Krieger, K. Kroeninger, H. Kroha, J. Kroll, J. Kroseberg, J. Krstic, U. Kruchonak, H. Krüger, T. Kruker, N. Krumnack, Z. V. Krumshteyn, M. K. Kruse, T. Kubota, S. Kuday, S. Kuehn, A. Kugel, T. Kuhl, V. Kukhtin, Y. Kulchitsky, S. Kuleshov, M. Kuna, J. Kunkle, A. Kupco, H. Kurashige, M. Kurata, Y. A. Kurochkin, V. Kus, E. S. Kuwertz, M. Kuze, J. Kvita, R. Kwee, A. La Rosa, L. La Rotonda, L. Labarga, S. Lablak, C. Lacasta, F. Lacava, J. Lacey, H. Lacker, D. Lacour, V. R. Lacuesta, E. Ladygin, R. Lafaye, B. Laforge, T. Lagouri, S. Lai, E. Laisne, L. Lambourne, C. L. Lampen, W. Lampl, E. Lancon, U. Landgraf, M. P. J. Landon, V. S. Lang, C. Lange, A. J. Lankford, F. Lanni, K. Lantzsch, A. Lanza, S. Laplace, C. Lapoire, J. F. Laporte, T. Lari, A. Larner, M. Lassnig, P. Laurelli, V. Lavorini, W. Lavrijsen, P. Laycock, O. Le Dortz, E. Le Guirriec, E. Le Menedeu, T. LeCompte, F. Ledroit-Guillon, H. Lee, J. S. H. Lee, S. C. Lee, L. Lee, M. Lefebvre, M. Legendre, F. Legger, C. Leggett, M. Lehmacher, G. Lehmann Miotto, A. G. Leister, M. A. L. Leite, R. Leitner, D. Lellouch, B. Lemmer, V. Lendermann, K. J. C. Leney, T. Lenz, G. Lenzen, B. Lenzi, K. Leonhardt, S. Leontsinis, F. Lepold, C. Leroy, J-R. Lessard, C. G. Lester, C. M. Lester, J. Levêque, D. Levin, L. J. Levinson, A. Lewis, G. H. Lewis, A. M. Leyko, M. Leyton, B. Li, B. Li, H. Li, H. L. Li, S. Li, X. Li, Z. Liang, H. Liao, B. Liberti, P. Lichard, K. Lie, W. Liebig, C. Limbach, A. Limosani, M. Limper, S. C. Lin, F. Linde, J. T. Linnemann, E. Lipeles, A. Lipniacka, T. M. Liss, D. Lissauer, A. Lister, A. M. Litke, D. Liu, J. B. Liu, L. Liu, M. Liu, Y. Liu, M. Livan, S. S. A. Livermore, A. Lleres, J. Llorente Merino, S. L. Lloyd, E. Lobodzinska, P. Loch, W. S. Lockman, T. Loddenkoetter, F. K. Loebinger, A. Loginov, C. W. Loh, T. Lohse, K. Lohwasser, M. Lokajicek, V. P. Lombardo, R. E. Long, L. Lopes, D. Lopez Mateos, J. Lorenz, N. Lorenzo Martinez, M. Losada, P. Loscutoff, F. Lo Sterzo, M. J. Losty, X. Lou, A. Lounis, K. F. Loureiro, J. Love, P. A. Love, A. J. Lowe, F. Lu, H. J. Lubatti, C. Luci, A. Lucotte, D. Ludwig, I. Ludwig, J. Ludwig, F. Luehring, G. Luijckx, W. Lukas, L. Luminari, E. Lund, B. Lund-Jensen, B. Lundberg, J. Lundberg, O. Lundberg, J. Lundquist, M. Lungwitz, D. Lynn, E. Lytken, H. Ma, L. L. Ma, G. Maccarrone, A. Macchiolo, B. Maček, J. Machado Miguens, D. Macina, R. Mackeprang, R. J. Madaras, H. J. Maddocks, W. F. Mader, A. K. Madsen, M. Maeno, T. Maeno, P. Mättig, S. Mättig, L. Magnoni, E. Magradze, K. Mahboubi, J. Mahlstedt, S. Mahmoud, G. Mahout, C. Maiani, C. Maidantchik, A. Maio, S. Majewski, Y. Makida, N. Makovec, P. Mal, B. Malaescu, Pa. Malecki, P. Malecki, V. P. Maleev, F. Malek, U. Mallik, D. Malon, C. Malone, S. Maltezos, V. Malyshev, S. Malyukov, J. Mamuzic, A. Manabe, L. Mandelli, I. Mandić, R. Mandrysch, J. Maneira, A. Manfredini, L. Manhaes de Andrade Filho, J. A. Manjarres Ramos, A. Mann, P. M. Manning, A. Manousakis-Katsikakis, B. Mansoulie, R. Mantifel, A. Mapelli, L. Mapelli, L. March, J. F. Marchand, F. Marchese, G. Marchiori, M. Marcisovsky, C. P. Marino, F. Marroquim, Z. Marshall, L. F. Marti, S. Marti-Garcia, B. Martin, B. Martin, J. P. Martin, T. A. Martin, V. J. Martin, B. Martin dit Latour, S. Martin-Haugh, H. Martinez, M. Martinez, V. Martinez Outschoorn, A. C. Martyniuk, M. Marx, F. Marzano, A. Marzin, L. Masetti, T. Mashimo, R. Mashinistov, J. Masik, A. L. Maslennikov, I. Massa, G. Massaro, N. Massol, P. Mastrandrea, A. Mastroberardino, T. Masubuchi, H. Matsunaga, T. Matsushita, C. Mattravers, J. Maurer, S. J. Maxfield, D. A. Maximov, R. Mazini, M. Mazur, L. Mazzaferro, M. Mazzanti, J. Mc Donald, S. P. Mc Kee, A. McCarn, R. L. McCarthy, T. G. McCarthy, N. A. McCubbin, K. W. McFarlane, J. A. Mcfayden, G. Mchedlidze, T. Mclaughlan, S. J. McMahon, R. A. McPherson, A. Meade, J. Mechnich, M. Mechtel, M. Medinnis, S. Meehan, R. Meera-Lebbai, T. Meguro, S. Mehlhase, A. Mehta, K. Meier, B. Meirose, C. Melachrinos, B. R. Mellado Garcia, F. Meloni, L. Mendoza Navas, Z. Meng, A. Mengarelli, S. Menke, E. Meoni, K. M. Mercurio, P. Mermod, L. Merola, C. Meroni, F. S. Merritt, H. Merritt, A. Messina, J. Metcalfe, A. S. Mete, C. Meyer, C. Meyer, J-P. Meyer, J. Meyer, J. Meyer, S. Michal, L. Micu, R. P. Middleton, S. Migas, L. Mijović, G. Mikenberg, M. Mikestikova, M. Mikuž, D. W. Miller, R. J. Miller, W. J. Mills, C. Mills, A. Milov, D. A. Milstead, D. Milstein, A. A. Minaenko, M. Miñano Moya, I. A. Minashvili, A. I. Mincer, B. Mindur, M. Mineev, Y. Ming, L. M. Mir, G. Mirabelli, J. Mitrevski, V. A. Mitsou, S. Mitsui, P. S. Miyagawa, J. U. Mjörnmark, T. Moa, V. Moeller, K. Mönig, N. Möser, S. Mohapatra, W. Mohr, R. Moles-Valls, A. Molfetas, J. Monk, E. Monnier, J. Montejo Berlingen, F. Monticelli, S. Monzani, R. W. Moore, G. F. Moorhead, C. Mora Herrera, A. Moraes, N. Morange, J. Morel, G. Morello, D. Moreno, M. Moreno Llácer, P. Morettini, M. Morgenstern, M. Morii, A. K. Morley, G. Mornacchi, J. D. Morris, L. Morvaj, H. G. Moser, M. Mosidze, J. Moss, R. Mount, E. Mountricha, S. V. Mouraviev, E. J. W. Moyse, F. Mueller, J. Mueller, K. Mueller, T. A. Müller, T. Mueller, D. Muenstermann, Y. Munwes, W. J. Murray, I. Mussche, E. Musto, A. G. Myagkov, M. Myska, O. Nackenhorst, J. Nadal, K. Nagai, R. Nagai, K. Nagano, A. Nagarkar, Y. Nagasaka, M. Nagel, A. M. Nairz, Y. Nakahama, K. Nakamura, T. Nakamura, I. Nakano, G. Nanava, A. Napier, R. Narayan, M. Nash, T. Nattermann, T. Naumann, G. Navarro, H. A. Neal, P. Yu. Nechaeva, T. J. Neep, A. Negri, G. Negri, M. Negrini, S. Nektarijevic, A. Nelson, T. K. Nelson, S. Nemecek, P. Nemethy, A. A. Nepomuceno, M. Nessi, M. S. Neubauer, M. Neumann, A. Neusiedl, R. M. Neves, P. Nevski, F. M. Newcomer, P. R. Newman, V. Nguyen Thi Hong, R. B. Nickerson, R. Nicolaidou, B. Nicquevert, F. Niedercorn, J. Nielsen, N. Nikiforou, A. Nikiforov, V. Nikolaenko, I. Nikolic-Audit, K. Nikolics, K. Nikolopoulos, H. Nilsen, P. Nilsson, Y. Ninomiya, A. Nisati, R. Nisius, T. Nobe, L. Nodulman, M. Nomachi, I. Nomidis, S. Norberg, M. Nordberg, J. Novakova, M. Nozaki, L. Nozka, A.-E. Nuncio-Quiroz, G. Nunes Hanninger, T. Nunnemann, E. Nurse, B. J. O’Brien, D. C. O’Neil, V. O’Shea, L. B. Oakes, F. G. Oakham, H. Oberlack, J. Ocariz, A. Ochi, S. Oda, S. Odaka, J. Odier, H. Ogren, A. Oh, S. H. Oh, C. C. Ohm, T. Ohshima, W. Okamura, H. Okawa, Y. Okumura, T. Okuyama, A. Olariu, A. G. Olchevski, S. A. Olivares Pino, M. Oliveira, D. Oliveira Damazio, E. Oliver Garcia, D. Olivito, A. Olszewski, J. Olszowska, A. Onofre, P. U. E. Onyisi, C. J. Oram, M. J. Oreglia, Y. Oren, D. Orestano, N. Orlando, C. Oropeza Barrera, R. S. Orr, B. Osculati, R. Ospanov, C. Osuna, G. Otero y Garzon, J. P. Ottersbach, M. Ouchrif, E. A. Ouellette, F. Ould-Saada, A. Ouraou, Q. Ouyang, A. Ovcharova, M. Owen, S. Owen, V. E. Ozcan, N. Ozturk, A. Pacheco Pages, C. Padilla Aranda, S. Pagan Griso, E. Paganis, C. Pahl, F. Paige, P. Pais, K. Pajchel, G. Palacino, C. P. Paleari, S. Palestini, D. Pallin, A. Palma, J. D. Palmer, Y. B. Pan, E. Panagiotopoulou, J. G. Panduro Vazquez, P. Pani, N. Panikashvili, S. Panitkin, D. Pantea, A. Papadelis, Th. D. Papadopoulou, A. Paramonov, D. Paredes Hernandez, W. Park, M. A. Parker, F. Parodi, J. A. Parsons, U. Parzefall, S. Pashapour, E. Pasqualucci, S. Passaggio, A. Passeri, F. Pastore, Fr. Pastore, G. Pásztor, S. Pataraia, N. D. Patel, J. R. Pater, S. Patricelli, T. Pauly, S. Pedraza Lopez, M. I. Pedraza Morales, S. V. Peleganchuk, D. Pelikan, H. Peng, B. Penning, A. Penson, J. Penwell, M. Perantoni, K. Perez, T. Perez Cavalcanti, E. Perez Codina, M. T. Pérez García-Estañ, V. Perez Reale, L. Perini, H. Pernegger, R. Perrino, P. Perrodo, V. D. Peshekhonov, K. Peters, B. A. Petersen, J. Petersen, T. C. Petersen, E. Petit, A. Petridis, C. Petridou, E. Petrolo, F. Petrucci, D. Petschull, M. Petteni, R. Pezoa, A. Phan, P. W. Phillips, G. Piacquadio, A. Picazio, E. Piccaro, M. Piccinini, S. M. Piec, R. Piegaia, D. T. Pignotti, J. E. Pilcher, A. D. Pilkington, J. Pina, M. Pinamonti, A. Pinder, J. L. Pinfold, A. Pingel, B. Pinto, C. Pizio, M.-A. Pleier, E. Plotnikova, A. Poblaguev, S. Poddar, F. Podlyski, L. Poggioli, D. Pohl, M. Pohl, G. Polesello, A. Policicchio, R. Polifka, A. Polini, J. Poll, V. Polychronakos, D. Pomeroy, K. Pommès, L. Pontecorvo, B. G. Pope, G. A. Popeneciu, D. S. Popovic, A. Poppleton, X. Portell Bueso, G. E. Pospelov, S. Pospisil, I. N. Potrap, C. J. Potter, C. T. Potter, G. Poulard, J. Poveda, V. Pozdnyakov, R. Prabhu, P. Pralavorio, A. Pranko, S. Prasad, R. Pravahan, S. Prell, K. Pretzl, D. Price, J. Price, L. E. Price, D. Prieur, M. Primavera, K. Prokofiev, F. Prokoshin, S. Protopopescu, J. Proudfoot, X. Prudent, M. Przybycien, H. Przysiezniak, S. Psoroulas, E. Ptacek, E. Pueschel, D. Puldon, J. Purdham, M. Purohit, P. Puzo, Y. Pylypchenko, J. Qian, A. Quadt, D. R. Quarrie, W. B. Quayle, M. Raas, V. Radeka, V. Radescu, P. Radloff, F. Ragusa, G. Rahal, A. M. Rahimi, D. Rahm, S. Rajagopalan, M. Rammensee, M. Rammes, A. S. Randle-Conde, K. Randrianarivony, K. Rao, F. Rauscher, T. C. Rave, M. Raymond, A. L. Read, D. M. Rebuzzi, A. Redelbach, G. Redlinger, R. Reece, K. Reeves, A. Reinsch, I. Reisinger, C. Rembser, Z. L. Ren, A. Renaud, M. Rescigno, S. Resconi, B. Resende, P. Reznicek, R. Rezvani, R. Richter, E. Richter-Was, M. Ridel, M. Rijssenbeek, A. Rimoldi, L. Rinaldi, R. R. Rios, E. Ritsch, I. Riu, G. Rivoltella, F. Rizatdinova, E. Rizvi, S. H. Robertson, A. Robichaud-Veronneau, D. Robinson, J. E. M. Robinson, A. Robson, J. G. Rocha de Lima, C. Roda, D. Roda Dos Santos, A. Roe, S. Roe, O. Røhne, S. Rolli, A. Romaniouk, M. Romano, G. Romeo, E. Romero Adam, N. Rompotis, L. Roos, E. Ros, S. Rosati, K. Rosbach, A. Rose, M. Rose, G. A. Rosenbaum, P. L. Rosendahl, O. Rosenthal, L. Rosselet, V. Rossetti, E. Rossi, L. P. Rossi, M. Rotaru, I. Roth, J. Rothberg, D. Rousseau, C. R. Royon, A. Rozanov, Y. Rozen, X. Ruan, F. Rubbo, I. Rubinskiy, N. Ruckstuhl, V. I. Rud, C. Rudolph, M. S. Rudolph, F. Rühr, A. Ruiz-Martinez, L. Rumyantsev, Z. Rurikova, N. A. Rusakovich, A. Ruschke, J. P. Rutherfoord, N. Ruthmann, P. Ruzicka, Y. F. Ryabov, M. Rybar, G. Rybkin, N. C. Ryder, A. F. Saavedra, I. Sadeh, H. F-W. Sadrozinski, R. Sadykov, F. Safai Tehrani, H. Sakamoto, G. Salamanna, A. Salamon, M. Saleem, D. Salek, D. Salihagic, A. Salnikov, J. Salt, B. M. Salvachua Ferrando, D. Salvatore, F. Salvatore, A. Salvucci, A. Salzburger, D. Sampsonidis, B. H. Samset, A. Sanchez, V. Sanchez Martinez, H. Sandaker, H. G. Sander, M. P. Sanders, M. Sandhoff, T. Sandoval, C. Sandoval, R. Sandstroem, D. P. C. Sankey, A. Sansoni, C. Santamarina Rios, C. Santoni, R. Santonico, H. Santos, I. Santoyo Castillo, J. G. Saraiva, T. Sarangi, E. Sarkisyan-Grinbaum, B. Sarrazin, F. Sarri, G. Sartisohn, O. Sasaki, Y. Sasaki, N. Sasao, I. Satsounkevitch, G. Sauvage, E. Sauvan, J. B. Sauvan, P. Savard, V. Savinov, D. O. Savu, L. Sawyer, D. H. Saxon, J. Saxon, C. Sbarra, A. Sbrizzi, D. A. Scannicchio, M. Scarcella, J. Schaarschmidt, P. Schacht, D. Schaefer, U. Schäfer, A. Schaelicke, S. Schaepe, S. Schaetzel, A. C. Schaffer, D. Schaile, R. D. Schamberger, V. Scharf, V. A. Schegelsky, D. Scheirich, M. Schernau, M. I. Scherzer, C. Schiavi, J. Schieck, M. Schioppa, S. Schlenker, E. Schmidt, K. Schmieden, C. Schmitt, S. Schmitt, B. Schneider, U. Schnoor, L. Schoeffel, A. Schoening, A. L. S. Schorlemmer, M. Schott, D. Schouten, J. Schovancova, M. Schram, C. Schroeder, N. Schroer, M. J. Schultens, J. Schultes, H.-C. Schultz-Coulon, H. Schulz, M. Schumacher, B. A. Schumm, Ph. Schune, A. Schwartzman, Ph. Schwegler, Ph. Schwemling, R. Schwienhorst, J. Schwindling, T. Schwindt, M. Schwoerer, F. G. Sciacca, E. Scifo, G. Sciolla, W. G. Scott, J. Searcy, G. Sedov, E. Sedykh, S. C. Seidel, A. Seiden, F. Seifert, J. M. Seixas, G. Sekhniaidze, S. J. Sekula, K. E. Selbach, D. M. Seliverstov, B. Sellden, G. Sellers, M. Seman, N. Semprini-Cesari, C. Serfon, L. Serin, L. Serkin, R. Seuster, H. Severini, A. Sfyrla, E. Shabalina, M. Shamim, L. Y. Shan, J. T. Shank, Q. T. Shao, M. Shapiro, P. B. Shatalov, K. Shaw, D. Sherman, P. Sherwood, S. Shimizu, M. Shimojima, T. Shin, M. Shiyakova, A. Shmeleva, M. J. Shochet, D. Short, S. Shrestha, E. Shulga, M. A. Shupe, P. Sicho, A. Sidoti, F. Siegert, Dj. Sijacki, O. Silbert, J. Silva, Y. Silver, D. Silverstein, S. B. Silverstein, V. Simak, O. Simard, Lj. Simic, S. Simion, E. Simioni, B. Simmons, R. Simoniello, M. Simonyan, P. Sinervo, N. B. Sinev, V. Sipica, G. Siragusa, A. Sircar, A. N. Sisakyan, S. Yu. Sivoklokov, J. Sjölin, T. B. Sjursen, L. A. Skinnari, H. P. Skottowe, K. Skovpen, P. Skubic, M. Slater, T. Slavicek, K. Sliwa, V. Smakhtin, B. H. Smart, L. Smestad, S. Yu. Smirnov, Y. Smirnov, L. N. Smirnova, O. Smirnova, B. C. Smith, K. M. Smith, M. Smizanska, K. Smolek, A. A. Snesarev, G. Snidero, S. W. Snow, J. Snow, S. Snyder, R. Sobie, J. Sodomka, A. Soffer, C. A. Solans, M. Solar, J. Solc, E. Yu. Soldatov, U. Soldevila, E. Solfaroli Camillocci, A. A. Solodkov, O. V. Solovyanov, V. Solovyev, N. Soni, A. Sood, V. Sopko, B. Sopko, M. Sosebee, R. Soualah, P. Soueid, A. Soukharev, D. South, S. Spagnolo, F. Spanò, R. Spighi, G. Spigo, R. Spiwoks, M. Spousta, T. Spreitzer, B. Spurlock, R. D. St. Denis, J. Stahlman, R. Stamen, E. Stanecka, R. W. Stanek, C. Stanescu, M. Stanescu-Bellu, M. M. Stanitzki, S. Stapnes, E. A. Starchenko, J. Stark, P. Staroba, P. Starovoitov, R. Staszewski, A. Staude, P. Stavina, G. Steele, P. Steinbach, P. Steinberg, I. Stekl, B. Stelzer, H. J. Stelzer, O. Stelzer-Chilton, H. Stenzel, S. Stern, G. A. Stewart, J. A. Stillings, M. C. Stockton, M. Stoebe, K. Stoerig, G. Stoicea, S. Stonjek, P. Strachota, A. R. Stradling, A. Straessner, J. Strandberg, S. Strandberg, A. Strandlie, M. Strang, E. Strauss, M. Strauss, P. Strizenec, R. Ströhmer, D. M. Strom, J. A. Strong, R. Stroynowski, B. Stugu, I. Stumer, J. Stupak, P. Sturm, N. A. Styles, D. A. Soh, D. Su, HS. Subramania, R. Subramaniam, A. Succurro, Y. Sugaya, C. Suhr, M. Suk, V. V. Sulin, S. Sultansoy, T. Sumida, X. Sun, J. E. Sundermann, K. Suruliz, G. Susinno, M. R. Sutton, Y. Suzuki, Y. Suzuki, M. Svatos, S. Swedish, I. Sykora, T. Sykora, J. Sánchez, D. Ta, K. Tackmann, A. Taffard, R. Tafirout, N. Taiblum, Y. Takahashi, H. Takai, R. Takashima, H. Takeda, T. Takeshita, Y. Takubo, M. Talby, A. Talyshev, M. C. Tamsett, K. G. Tan, J. Tanaka, R. Tanaka, S. Tanaka, S. Tanaka, A. J. Tanasijczuk, K. Tani, N. Tannoury, S. Tapprogge, D. Tardif, S. Tarem, F. Tarrade, G. F. Tartarelli, P. Tas, M. Tasevsky, E. Tassi, Y. Tayalati, C. Taylor, F. E. Taylor, G. N. Taylor, W. Taylor, M. Teinturier, F. A. Teischinger, M. Teixeira Dias Castanheira, P. Teixeira-Dias, K. K. Temming, H. Ten Kate, P. K. Teng, S. Terada, K. Terashi, J. Terron, M. Testa, R. J. Teuscher, J. Therhaag, T. Theveneaux-Pelzer, S. Thoma, J. P. Thomas, E. N. Thompson, P. D. Thompson, P. D. Thompson, A. S. Thompson, L. A. Thomsen, E. Thomson, M. Thomson, W. M. Thong, R. P. Thun, F. Tian, M. J. Tibbetts, T. Tic, V. O. Tikhomirov, Y. A. Tikhonov, S. Timoshenko, E. Tiouchichine, P. Tipton, S. Tisserant, T. Todorov, S. Todorova-Nova, B. Toggerson, J. Tojo, S. Tokár, K. Tokushuku, K. Tollefson, M. Tomoto, L. Tompkins, K. Toms, A. Tonoyan, C. Topfel, N. D. Topilin, E. Torrence, H. Torres, E. Torró Pastor, J. Toth, F. Touchard, D. R. Tovey, T. Trefzger, L. Tremblet, A. Tricoli, I. M. Trigger, S. Trincaz-Duvoid, M. F. Tripiana, N. Triplett, W. Trischuk, B. Trocmé, C. Troncon, M. Trottier-McDonald, P. True, M. Trzebinski, A. Trzupek, C. Tsarouchas, J. C-L. Tseng, M. Tsiakiris, P. V. Tsiareshka, D. Tsionou, G. Tsipolitis, S. Tsiskaridze, V. Tsiskaridze, E. G. Tskhadadze, I. I. Tsukerman, V. Tsulaia, J.-W. Tsung, S. Tsuno, D. Tsybychev, A. Tua, A. Tudorache, V. Tudorache, J. M. Tuggle, M. Turala, D. Turecek, I. Turk Cakir, R. Turra, P. M. Tuts, A. Tykhonov, M. Tylmad, M. Tyndel, G. Tzanakos, K. Uchida, I. Ueda, R. Ueno, M. Ughetto, M. Ugland, M. Uhlenbrock, F. Ukegawa, G. Unal, A. Undrus, G. Unel, F. C. Ungaro, Y. Unno, D. Urbaniec, P. Urquijo, G. Usai, L. Vacavant, V. Vacek, B. Vachon, S. Vahsen, S. Valentinetti, A. Valero, L. Valery, S. Valkar, E. Valladolid Gallego, S. Vallecorsa, J. A. Valls Ferrer, R. Van Berg, P. C. Van Der Deijl, R. van der Geer, H. van der Graaf, R. Van Der Leeuw, E. van der Poel, D. van der Ster, N. van Eldik, P. van Gemmeren, J. Van Nieuwkoop, I. van Vulpen, M. Vanadia, W. Vandelli, A. Vaniachine, P. Vankov, F. Vannucci, R. Vari, E. W. Varnes, T. Varol, D. Varouchas, A. Vartapetian, K. E. Varvell, V. I. Vassilakopoulos, F. Vazeille, T. Vazquez Schroeder, G. Vegni, J. J. Veillet, F. Veloso, R. Veness, S. Veneziano, A. Ventura, D. Ventura, M. Venturi, N. Venturi, V. Vercesi, M. Verducci, W. Verkerke, J. C. Vermeulen, A. Vest, M. C. Vetterli, I. Vichou, T. Vickey, O. E. Vickey Boeriu, G. H. A. Viehhauser, S. Viel, M. Villa, M. Villaplana Perez, E. Vilucchi, M. G. Vincter, E. Vinek, V. B. Vinogradov, M. Virchaux, J. Virzi, O. Vitells, M. Viti, I. Vivarelli, F. Vives Vaque, S. Vlachos, D. Vladoiu, M. Vlasak, A. Vogel, P. Vokac, G. Volpi, M. Volpi, G. Volpini, H. von der Schmitt, H. von Radziewski, E. von Toerne, V. Vorobel, V. Vorwerk, M. Vos, R. Voss, J. H. Vossebeld, N. Vranjes, M. Vranjes Milosavljevic, V. Vrba, M. Vreeswijk, T. Vu Anh, R. Vuillermet, I. Vukotic, W. Wagner, P. Wagner, H. Wahlen, S. Wahrmund, J. Wakabayashi, S. Walch, J. Walder, R. Walker, W. Walkowiak, R. Wall, P. Waller, B. Walsh, C. Wang, H. Wang, H. Wang, J. Wang, J. Wang, R. Wang, S. M. Wang, T. Wang, A. Warburton, C. P. Ward, D. R. Wardrope, M. Warsinsky, A. Washbrook, C. Wasicki, I. Watanabe, P. M. Watkins, A. T. Watson, I. J. Watson, M. F. Watson, G. Watts, S. Watts, A. T. Waugh, B. M. Waugh, M. S. Weber, J. S. Webster, A. R. Weidberg, P. Weigell, J. Weingarten, C. Weiser, P. S. Wells, T. Wenaus, D. Wendland, Z. Weng, T. Wengler, S. Wenig, N. Wermes, M. Werner, P. Werner, M. Werth, M. Wessels, J. Wetter, C. Weydert, K. Whalen, A. White, M. J. White, S. White, S. R. Whitehead, D. Whiteson, D. Whittington, D. Wicke, F. J. Wickens, W. Wiedenmann, M. Wielers, P. Wienemann, C. Wiglesworth, L. A. M. Wiik-Fuchs, P. A. Wijeratne, A. Wildauer, M. A. Wildt, I. Wilhelm, H. G. Wilkens, J. Z. Will, E. Williams, H. H. Williams, S. Williams, W. Willis, S. Willocq, J. A. Wilson, M. G. Wilson, A. Wilson, I. Wingerter-Seez, S. Winkelmann, F. Winklmeier, M. Wittgen, S. J. Wollstadt, M. W. Wolter, H. Wolters, W. C. Wong, G. Wooden, B. K. Wosiek, J. Wotschack, M. J. Woudstra, K. W. Wozniak, K. Wraight, M. Wright, B. Wrona, S. L. Wu, X. Wu, Y. Wu, E. Wulf, B. M. Wynne, S. Xella, M. Xiao, S. Xie, C. Xu, D. Xu, L. Xu, B. Yabsley, S. Yacoob, M. Yamada, H. Yamaguchi, A. Yamamoto, K. Yamamoto, S. Yamamoto, T. Yamamura, T. Yamanaka, K. Yamauchi, T. Yamazaki, Y. Yamazaki, Z. Yan, H. Yang, H. Yang, U. K. Yang, Y. Yang, Z. Yang, S. Yanush, L. Yao, Y. Yasu, E. Yatsenko, J. Ye, S. Ye, A. L. Yen, M. Yilmaz, R. Yoosoofmiya, K. Yorita, R. Yoshida, K. Yoshihara, C. Young, C. J. Young, S. Youssef, D. Yu, D. R. Yu, J. Yu, J. Yu, L. Yuan, A. Yurkewicz, B. Zabinski, R. Zaidan, A. M. Zaitsev, L. Zanello, D. Zanzi, A. Zaytsev, C. Zeitnitz, M. Zeman, A. Zemla, O. Zenin, T. Ženiš, Z. Zinonos, D. Zerwas, G. Zevi della Porta, D. Zhang, H. Zhang, J. Zhang, X. Zhang, Z. Zhang, L. Zhao, Z. Zhao, A. Zhemchugov, J. Zhong, B. Zhou, N. Zhou, Y. Zhou, C. G. Zhu, H. Zhu, J. Zhu, Y. Zhu, X. Zhuang, V. Zhuravlov, A. Zibell, D. Zieminska, N. I. Zimin, R. Zimmermann, S. Zimmermann, S. Zimmermann, M. Ziolkowski, R. Zitoun, L. Živković, V. V. Zmouchko, G. Zobernig, A. Zoccoli, M. zur Nedden, V. Zutshi, L. Zwalinski

**Affiliations:** 1 CERN, 1211 Geneva 23, Switzerland; 2 School of Chemistry and Physics, University of Adelaide, Adelaide, Australia; 3 Physics Department, SUNY Albany, Albany, NY United States of America; 4 Department of Physics, University of Alberta, Edmonton, AB Canada; 5 Department of Physics, Ankara University, Ankara, Turkey; 6 Department of Physics, Dumlupinar University, Kutahya, Turkey; 7 Department of Physics, Gazi University, Ankara, Turkey; 8 Division of Physics, TOBB University of Economics and Technology, Ankara, Turkey; 9 Turkish Atomic Energy Authority, Ankara, Turkey; 10 LAPP, CNRS/IN2P3 and Université de Savoie, Annecy-le-Vieux, France; 11 High Energy Physics Division, Argonne National Laboratory, Argonne, IL United States of America; 12 Department of Physics, University of Arizona, Tucson, AZ United States of America; 13 Department of Physics, The University of Texas at Arlington, Arlington, TX United States of America; 14 Physics Department, University of Athens, Athens, Greece; 15 Physics Department, National Technical University of Athens, Zografou, Greece; 16 Institute of Physics, Azerbaijan Academy of Sciences, Baku, Azerbaijan; 17 Institut de Física d’Altes Energies and Departament de Física de la Universitat Autònoma de Barcelona and ICREA, Barcelona, Spain; 18 Institute of Physics, University of Belgrade, Belgrade, Serbia; 19 Vinca Institute of Nuclear Sciences, University of Belgrade, Belgrade, Serbia; 20 Department for Physics and Technology, University of Bergen, Bergen, Norway; 21 Physics Division, Lawrence Berkeley National Laboratory and University of California, Berkeley, CA United States of America; 22 Department of Physics, Humboldt University, Berlin, Germany; 23 Albert Einstein Center for Fundamental Physics and Laboratory for High Energy Physics, University of Bern, Bern, Switzerland; 24 School of Physics and Astronomy, University of Birmingham, Birmingham, United Kingdom; 25 Department of Physics, Bogazici University, Istanbul, Turkey; 26 Division of Physics, Dogus University, Istanbul, Turkey; 27 Department of Physics Engineering, Gaziantep University, Gaziantep, Turkey; 28 Department of Physics, Istanbul Technical University, Istanbul, Turkey; 29 INFN Sezione di Bologna, Bologna, Italy; 30 Dipartimento di Fisica, Università di Bologna, Bologna, Italy; 31 Physikalisches Institut, University of Bonn, Bonn, Germany; 32 Department of Physics, Boston University, Boston, MA United States of America; 33 Department of Physics, Brandeis University, Waltham, MA United States of America; 34 Universidade Federal do Rio De Janeiro COPPE/EE/IF, Rio de Janeiro, Brazil; 35 Federal University of Juiz de Fora (UFJF), Juiz de Fora, Brazil; 36 Federal University of Sao Joao del Rei (UFSJ), Sao Joao del Rei, Brazil; 37 Instituto de Fisica, Universidade de Sao Paulo, Sao Paulo, Brazil; 38 Physics Department, Brookhaven National Laboratory, Upton, NY United States of America; 39 National Institute of Physics and Nuclear Engineering, Bucharest, Romania; 40 University Politehnica Bucharest, Bucharest, Romania; 41 West University in Timisoara, Timisoara, Romania; 42 Departamento de Física, Universidad de Buenos Aires, Buenos Aires, Argentina; 43 Cavendish Laboratory, University of Cambridge, Cambridge, United Kingdom; 44 Department of Physics, Carleton University, Ottawa, ON Canada; 45 CERN, Geneva, Switzerland; 46 Enrico Fermi Institute, University of Chicago, Chicago, IL United States of America; 47 Departamento de Física, Pontificia Universidad Católica de Chile, Santiago, Chile; 48 Departamento de Física, Universidad Técnica Federico Santa María, Valparaíso, Chile; 49 Institute of High Energy Physics, Chinese Academy of Sciences, Beijing, China; 50 Department of Modern Physics, University of Science and Technology of China, Anhui, China; 51 Department of Physics, Nanjing University, Jiangsu, China; 52 School of Physics, Shandong University, Shandong, China; 53 Physics Department, Shanghai Jiao Tong University, Shanghai, China; 54 Laboratoire de Physique Corpusculaire, Clermont Université and Université Blaise Pascal and CNRS/IN2P3, Clermont-Ferrand, France; 55 Nevis Laboratory, Columbia University, Irvington, NY United States of America; 56 Niels Bohr Institute, University of Copenhagen, Kobenhavn, Denmark; 57 INFN Gruppo Collegato di Cosenza, Arcavata di Rende, Italy; 58 Dipartimento di Fisica, Università della Calabria, Arcavata di Rende, Italy; 59 Faculty of Physics and Applied Computer Science, AGH University of Science and Technology, Krakow, Poland; 60 The Henryk Niewodniczanski Institute of Nuclear Physics, Polish Academy of Sciences, Krakow, Poland; 61 Physics Department, Southern Methodist University, Dallas, TX United States of America; 62 Physics Department, University of Texas at Dallas, Richardson, TX United States of America; 63 DESY, Hamburg and Zeuthen, Germany; 64 Institut für Experimentelle Physik IV, Technische Universität Dortmund, Dortmund, Germany; 65 Institut für Kern- und Teilchenphysik, Technical University Dresden, Dresden, Germany; 66 Department of Physics, Duke University, Durham, NC United States of America; 67 SUPA - School of Physics and Astronomy, University of Edinburgh, Edinburgh, United Kingdom; 68 INFN Laboratori Nazionali di Frascati, Frascati, Italy; 69 Fakultät für Mathematik und Physik, Albert-Ludwigs-Universität, Freiburg, Germany; 70 Section de Physique, Université de Genève, Geneva, Switzerland; 71 INFN Sezione di Genova, Genova, Italy; 72 Dipartimento di Fisica, Università di Genova, Genova, Italy; 73 E. Andronikashvili Institute of Physics, Iv. Javakhishvili Tbilisi State University, Tbilisi, Georgia; 74 High Energy Physics Institute, Tbilisi State University, Tbilisi, Georgia; 75 II Physikalisches Institut, Justus-Liebig-Universität Giessen, Giessen, Germany; 76 SUPA - School of Physics and Astronomy, University of Glasgow, Glasgow, United Kingdom; 77 II Physikalisches Institut, Georg-August-Universität, Göttingen, Germany; 78 Laboratoire de Physique Subatomique et de Cosmologie, Université Joseph Fourier and CNRS/IN2P3 and Institut National Polytechnique de Grenoble, Grenoble, France; 79 Department of Physics, Hampton University, Hampton, VA United States of America; 80 Laboratory for Particle Physics and Cosmology, Harvard University, Cambridge, MA United States of America; 81 Kirchhoff-Institut für Physik, Ruprecht-Karls-Universität Heidelberg, Heidelberg, Germany; 82 Physikalisches Institut, Ruprecht-Karls-Universität Heidelberg, Heidelberg, Germany; 83 ZITI Institut für technische Informatik, Ruprecht-Karls-Universität Heidelberg, Mannheim, Germany; 84 Faculty of Applied Information Science, Hiroshima Institute of Technology, Hiroshima, Japan; 85 Department of Physics, Indiana University, Bloomington, IN United States of America; 86 Institut für Astro- und Teilchenphysik, Leopold-Franzens-Universität, Innsbruck, Austria; 87 University of Iowa, Iowa City, IA United States of America; 88 Department of Physics and Astronomy, Iowa State University, Ames, IA United States of America; 89 Joint Institute for Nuclear Research, JINR Dubna, Dubna, Russia; 90 KEK, High Energy Accelerator Research Organization, Tsukuba, Japan; 91 Graduate School of Science, Kobe University, Kobe, Japan; 92 Faculty of Science, Kyoto University, Kyoto, Japan; 93 Kyoto University of Education, Kyoto, Japan; 94 Department of Physics, Kyushu University, Fukuoka, Japan; 95 Instituto de Física La Plata, Universidad Nacional de La Plata and CONICET, La Plata, Argentina; 96 Physics Department, Lancaster University, Lancaster, United Kingdom; 97 INFN Sezione di Lecce, Lecce, Italy; 98 Dipartimento di Matematica e Fisica, Università del Salento, Lecce, Italy; 99 Oliver Lodge Laboratory, University of Liverpool, Liverpool, United Kingdom; 100 Department of Physics, Jožef Stefan Institute and University of Ljubljana, Ljubljana, Slovenia; 101 School of Physics and Astronomy, Queen Mary University of London, London, United Kingdom; 102 Department of Physics, Royal Holloway University of London, Surrey, United Kingdom; 103 Department of Physics and Astronomy, University College London, London, United Kingdom; 104 Laboratoire de Physique Nucléaire et de Hautes Energies, UPMC and Université Paris-Diderot and CNRS/IN2P3, Paris, France; 105 Fysiska institutionen, Lunds universitet, Lund, Sweden; 106 Departamento de Fisica Teorica C-15, Universidad Autonoma de Madrid, Madrid, Spain; 107 Institut für Physik, Universität Mainz, Mainz, Germany; 108 School of Physics and Astronomy, University of Manchester, Manchester, United Kingdom; 109 CPPM, Aix-Marseille Université and CNRS/IN2P3, Marseille, France; 110 Department of Physics, University of Massachusetts, Amherst, MA United States of America; 111 Department of Physics, McGill University, Montreal, QC Canada; 112 School of Physics, University of Melbourne, Victoria, Australia; 113 Department of Physics, The University of Michigan, Ann Arbor, MI United States of America; 114 Department of Physics and Astronomy, Michigan State University, East Lansing, MI United States of America; 115 INFN Sezione di Milano, Milano, Italy; 116 Dipartimento di Fisica, Università di Milano, Milano, Italy; 117 B.I. Stepanov Institute of Physics, National Academy of Sciences of Belarus, Minsk, Republic of Belarus; 118 National Scientific and Educational Centre for Particle and High Energy Physics, Minsk, Republic of Belarus; 119 Department of Physics, Massachusetts Institute of Technology, Cambridge, MA United States of America; 120 Group of Particle Physics, University of Montreal, Montreal, QC Canada; 121 P.N. Lebedev Institute of Physics, Academy of Sciences, Moscow, Russia; 122 Institute for Theoretical and Experimental Physics (ITEP), Moscow, Russia; 123 Moscow Engineering and Physics Institute (MEPhI), Moscow, Russia; 124 D.V.Skobeltsyn Institute of Nuclear Physics, M.V.Lomonosov Moscow State University, Moscow, Russia; 125 Fakultät für Physik, Ludwig-Maximilians-Universität München, München, Germany; 126 Max-Planck-Institut für Physik (Werner-Heisenberg-Institut), München, Germany; 127 Nagasaki Institute of Applied Science, Nagasaki, Japan; 128 Graduate School of Science and Kobayashi-Maskawa Institute, Nagoya University, Nagoya, Japan; 129 INFN Sezione di Napoli, Napoli, Italy; 130 Dipartimento di Scienze Fisiche, Università di Napoli, Napoli, Italy; 131 Department of Physics and Astronomy, University of New Mexico, Albuquerque, NM United States of America; 132 Institute for Mathematics, Astrophysics and Particle Physics, Radboud University Nijmegen/Nikhef, Nijmegen, Netherlands; 133 Nikhef National Institute for Subatomic Physics and University of Amsterdam, Amsterdam, Netherlands; 134 Department of Physics, Northern Illinois University, DeKalb, IL United States of America; 135 Budker Institute of Nuclear Physics, SB RAS, Novosibirsk, Russia; 136 Department of Physics, New York University, New York, NY United States of America; 137 Ohio State University, Columbus, OH United States of America; 138 Faculty of Science, Okayama University, Okayama, Japan; 139 Homer L. Dodge Department of Physics and Astronomy, University of Oklahoma, Norman, OK United States of America; 140 Department of Physics, Oklahoma State University, Stillwater, OK United States of America; 141 RCPTM, Palacký University, Olomouc, Czech Republic; 142 Center for High Energy Physics, University of Oregon, Eugene, OR United States of America; 143 LAL, Université Paris-Sud and CNRS/IN2P3, Orsay, France; 144 Graduate School of Science, Osaka University, Osaka, Japan; 145 Department of Physics, University of Oslo, Oslo, Norway; 146 Department of Physics, Oxford University, Oxford, United Kingdom; 147 INFN Sezione di Pavia, Pavia, Italy; 148 Dipartimento di Fisica, Università di Pavia, Pavia, Italy; 149 Department of Physics, University of Pennsylvania, Philadelphia, PA United States of America; 150 Petersburg Nuclear Physics Institute, Gatchina, Russia; 151 INFN Sezione di Pisa, Pisa, Italy; 152 Dipartimento di Fisica E. Fermi, Università di Pisa, Pisa, Italy; 153 Department of Physics and Astronomy, University of Pittsburgh, Pittsburgh, PA United States of America; 154 Laboratorio de Instrumentacao e Fisica Experimental de Particulas - LIP, Lisboa, Portugal; 155 Departamento de Fisica Teorica y del Cosmos and CAFPE, Universidad de Granada, Granada, Spain; 156 Institute of Physics, Academy of Sciences of the Czech Republic, Praha, Czech Republic; 157 Czech Technical University in Prague, Praha, Czech Republic; 158 Faculty of Mathematics and Physics, Charles University in Prague, Praha, Czech Republic; 159 State Research Center Institute for High Energy Physics, Protvino, Russia; 160 Particle Physics Department, Rutherford Appleton Laboratory, Didcot, United Kingdom; 161 Physics Department, University of Regina, Regina, SK Canada; 162 Ritsumeikan University, Kusatsu, Shiga Japan; 163 INFN Sezione di Roma I, Roma, Italy; 164 Dipartimento di Fisica, Università La Sapienza, Roma, Italy; 165 INFN Sezione di Roma Tor Vergata, Roma, Italy; 166 Dipartimento di Fisica, Università di Roma Tor Vergata, Roma, Italy; 167 INFN Sezione di Roma Tre, Roma, Italy; 168 Dipartimento di Fisica, Università Roma Tre, Roma, Italy; 169 Faculté des Sciences Ain Chock, Réseau Universitaire de Physique des Hautes Energies - Université Hassan II, Casablanca, Morocco; 170 Centre National de l’Energie des Sciences Techniques Nucleaires, Rabat, Morocco; 171 Faculté des Sciences Semlalia, Université Cadi Ayyad, LPHEA-Marrakech, Morocco; 172 Faculté des Sciences, Université Mohamed Premier and LPTPM, Oujda, Morocco; 173 Faculté des sciences, Université Mohammed V-Agdal, Rabat, Morocco; 174 DSM/IRFU (Institut de Recherches sur les Lois Fondamentales de l’Univers), CEA Saclay (Commissariat à l’Energie Atomique et aux Energies Alternatives), Gif-sur-Yvette, France; 175 Santa Cruz Institute for Particle Physics, University of California Santa Cruz, Santa Cruz, CA United States of America; 176 Department of Physics, University of Washington, Seattle, WA United States of America; 177 Department of Physics and Astronomy, University of Sheffield, Sheffield, United Kingdom; 178 Department of Physics, Shinshu University, Nagano, Japan; 179 Fachbereich Physik, Universität Siegen, Siegen, Germany; 180 Department of Physics, Simon Fraser University, Burnaby, BC Canada; 181 SLAC National Accelerator Laboratory, Stanford, CA United States of America; 182 Faculty of Mathematics, Physics & Informatics, Comenius University, Bratislava, Slovak Republic; 183 Department of Subnuclear Physics, Institute of Experimental Physics of the Slovak Academy of Sciences, Kosice, Slovak Republic; 184 Department of Physics, University of Johannesburg, Johannesburg, South Africa; 185 School of Physics, University of the Witwatersrand, Johannesburg, South Africa; 186 Department of Physics, Stockholm University, Stockholm, Sweden; 187 The Oskar Klein Centre, Stockholm, Sweden; 188 Physics Department, Royal Institute of Technology, Stockholm, Sweden; 189 Departments of Physics & Astronomy and Chemistry, Stony Brook University, Stony Brook, NY United States of America; 190 Department of Physics and Astronomy, University of Sussex, Brighton, United Kingdom; 191 School of Physics, University of Sydney, Sydney, Australia; 192 Institute of Physics, Academia Sinica, Taipei, Taiwan; 193 Department of Physics, Technion: Israel Institute of Technology, Haifa, Israel; 194 Raymond and Beverly Sackler School of Physics and Astronomy, Tel Aviv University, Tel Aviv, Israel; 195 Department of Physics, Aristotle University of Thessaloniki, Thessaloniki, Greece; 196 International Center for Elementary Particle Physics and Department of Physics, The University of Tokyo, Tokyo, Japan; 197 Graduate School of Science and Technology, Tokyo Metropolitan University, Tokyo, Japan; 198 Department of Physics, Tokyo Institute of Technology, Tokyo, Japan; 199 Department of Physics, University of Toronto, Toronto, ON Canada; 200 TRIUMF, Vancouver, BC Canada; 201 Department of Physics and Astronomy, York University, Toronto, ON Canada; 202 Faculty of Pure and Applied Sciences, University of Tsukuba, Tsukuba, Japan; 203 Department of Physics and Astronomy, Tufts University, Medford, MA United States of America; 204 Centro de Investigaciones, Universidad Antonio Narino, Bogota, Colombia; 205 Department of Physics and Astronomy, University of California Irvine, Irvine, CA United States of America; 206 INFN Gruppo Collegato di Udine, Udine, Italy; 207 ICTP, Trieste, Italy; 208 Dipartimento di Chimica, Fisica e Ambiente, Università di Udine, Udine, Italy; 209 Department of Physics, University of Illinois, Urbana, IL United States of America; 210 Department of Physics and Astronomy, University of Uppsala, Uppsala, Sweden; 211 Instituto de Física Corpuscular (IFIC) and Departamento de Física Atómica, Molecular y Nuclear and Departamento de Ingeniería Electrónica and Instituto de Microelectrónica de Barcelona (IMB-CNM), University of Valencia and CSIC, Valencia, Spain; 212 Department of Physics, University of British Columbia, Vancouver, BC Canada; 213 Department of Physics and Astronomy, University of Victoria, Victoria, BC Canada; 214 Department of Physics, University of Warwick, Coventry, United Kingdom; 215 Waseda University, Tokyo, Japan; 216 Department of Particle Physics, The Weizmann Institute of Science, Rehovot, Israel; 217 Department of Physics, University of Wisconsin, Madison, WI United States of America; 218 Fakultät für Physik und Astronomie, Julius-Maximilians-Universität, Würzburg, Germany; 219 Fachbereich C Physik, Bergische Universität Wuppertal, Wuppertal, Germany; 220 Department of Physics, Yale University, New Haven, CT United States of America; 221 Yerevan Physics Institute, Yerevan, Armenia; 222 Centre de Calcul de l’Institut National de Physique Nucléaire et de Physique des Particules (IN2P3), Villeurbanne, France

## Abstract

A search for a charged Higgs boson (*H* ^+^) in $t\bar{t}$ decays is presented, where one of the top quarks decays via *t*→*H* ^+^ *b*, followed by *H* ^+^→ two jets ($c\bar{s}$). The other top quark decays to *Wb*, where the *W* boson then decays into a lepton (*e*/*μ*) and a neutrino. The data were recorded in *pp* collisions at $\sqrt{s} = 7~\mathrm {TeV}$ by the ATLAS detector at the LHC in 2011, and correspond to an integrated luminosity of 4.7 fb^−1^. With no observation of a signal, 95 % confidence level (CL) upper limits are set on the decay branching ratio of top quarks to charged Higgs bosons varying between 5 % and 1 % for *H* ^+^ masses between 90 GeV and 150 GeV, assuming $\mathcal{B}(H^{+} \rightarrow c\bar{s})=100~\%$.

## Introduction

In the Standard Model (SM), electroweak symmetry breaking (EWSB) occurs through a single complex scalar doublet field and results in a single physical state, the Higgs boson [1–3]. A particle with characteristics of the SM Higgs boson has been discovered by both ATLAS [4] and CMS [5]. Beyond the SM, many models have been proposed, extending the Higgs sector to explain EWSB. The newly discovered boson is compatible with many of these models so that discovering its true nature is crucial to understanding EWSB. Two Higgs-doublet models (2HDM) [6] are simple extensions of the SM with five observable Higgs bosons, of which two are charged (*H* ^+^ and *H* ^−^) and three are neutral (*h* ^0^, *H* ^0^ and *A* ^0^). The discovery of a charged Higgs boson would be a signal for new physics beyond the SM.

The Minimal Supersymmetric Standard Model (MSSM) [7] is an example of a 2HDM. At tree level, the MSSM Higgs sector is determined by two independent parameters, which can be taken to be the mass $m_{H^{+}}$ and the ratio of the two Higgs doublet vacuum expectation values, parametrised by tan*β*. In the MSSM, a light *H* ^+^ (defined as $m_{H^{+}}<m_{t}$) decays predominantly to $c\bar{s}$, $b\bar{b}W^{+}$, and *τ* ^+^ *ν*, with the respective branching ratios depending on tan*β* and $m_{H^{+}}$. Charge conjugated processes are implied throughout this paper. For tan*β*<1, $c\bar{s}$ is an important decay mode with $\mathcal{B}(H^{+} \rightarrow c\bar{s})$ near 70 % [8, 9] for $m_{H^{\pm}} \simeq110~\mathrm{GeV}$, whereas for tan*β*>3, *H* ^+^→*τ* ^+^ *ν* dominates (90 %). For higher *H* ^+^ masses at low tan*β*, the decay mode $H^{+}\rightarrow Wb\bar{b}$ can be dominant. A light MSSM charged Higgs boson is viable at a relatively low tan*β*≈6 in certain MSSM benchmark scenarios [10] that take into account the discovery of a Higgs boson with a mass of 125 GeV at the LHC.

The LEP experiments placed lower limits on $m_{H^{+}}$ in any type-II 2HDM [11] varying between 75 GeV and 91 GeV [12–16] depending on the assumed decay branching ratios for the charged Higgs boson. At the Tevatron, searches for charged Higgs bosons have been extended to larger values of $m_{H^{+}}$. No evidence for a *H* ^+^ was found and upper limits were set on the branching ratio $\mathcal{B}(t \rightarrow H^{+}b)$ varying between 10 % and 30 % for a light *H* ^+^ under the assumption of $\mathcal{B}(H^{+} \rightarrow c\bar {s})=100~\%$ [17, 18]. The discovery of a Higgs boson at the LHC is a weak constraint on many 2HDMs, and is compatible with the existence of a light charged Higgs decaying to two jets, especially in type I 2HDMs [19, 20].

In this paper, a search for a charged Higgs boson produced in $t\bar{t}$ decays is presented, where one of the top quarks decays via *t*→*H* ^+^ *b* with the charged Higgs boson subsequently decaying to two jets ($c\bar{s} $), where again a 100 % branching fraction is assumed. The other top quark decays according to the SM via $\bar{t} \rightarrow W^{-}\bar{b}$ with the *W* boson decaying into a lepton (*e*/*μ*) and the corresponding neutrino. The signal process therefore has the same topology as SM $t\bar{t}$ decays in the lepton+jets channel, where one *W* decays to two jets and the other to a lepton and corresponding neutrino, but the invariant mass of the two jets from the *H* ^+^ peaks at $m_{H^{+}}$. The search is performed by comparing the dijet mass spectrum in the data with the prediction from SM top-quark decays and with the expectation of a top quark having a non-zero branching ratio for decay to *H* ^+^ *b*.

## Detector description and event samples

The data used in the analysis were recorded by the ATLAS detector in proton–proton (*pp*) collisions at a centre-of-mass energy of $\sqrt {s}=7~\mathrm{TeV}$ during the 2011 data-taking period of the Large Hadron Collider (LHC) [21]. Events were required to pass a high-transverse momentum (*p*_T_) single-lepton (*e*/*μ*) trigger, and to have been recorded when all detector systems critical to muon, electron, and jet identification were operational. The lepton triggers required in the different data taking periods had varying *p*_T_ thresholds: 20–22 GeV for the electron trigger and 18 GeV for the muon trigger. The resulting dataset corresponds to an integrated luminosity of 4.7 fb^−1^ [22, 23].

The ATLAS detector [24] consists of an inner tracking system immersed in a 2 T axial magnetic field provided by a thin solenoid; electromagnetic and hadronic calorimeters; and a muon spectrometer (MS) embedded in a toroidal magnet system. The inner detector tracking system (ID) comprises a silicon pixel detector closest to the beamline, a silicon microstrip detector, and a straw tube transition radiation tracker. The electromagnetic (EM) calorimeters are high-granularity liquid-argon sampling calorimeters with lead as the absorber material in the barrel and endcap regions, and copper in the forward region. The hadronic calorimetry uses two different detector technologies. The barrel calorimeter (|*η*|<1.7)^1^ consists of scintillator tiles interleaved with steel absorber plates. The endcap (1.5<|*η*|<3.2) and forward (3.1<|*η*|<4.9) calorimeters both use liquid argon as the active material, and copper and tungsten respectively as the absorber. The MS consists of three large superconducting toroids each with eight coils, and a system of precision tracking and fast trigger chambers.

The largest background to the charged Higgs boson signal is the SM production and decay of $t\bar{t}$ pairs. Additional background contributions (referred to as non-$t\bar{t}$ backgrounds) arise from the production of a single top quark, of a *W* or *Z* boson with additional jets, of QCD multi-jets, and of dibosons.

Top-quark pair and single top-quark events (*Wt*-channel and *s*-channel) were generated using the mc@n-lo 4.01 [25–28] Monte Carlo (MC) generator coupled to Herwig 6.520.2 [29] to provide the parton showering and hadronisation using the AUET2-CT10 [30, 31] tune; Jimmy [32] was used to model the underlying event. Single top-quark events in the *t*-channel were generated using AcerMC 3.8 [33] coupled to Pythia 6.425 [34] with the AUET2-MRST2007LO** [30, 35] tune. *W*/*Z*+jet and diboson events were generated using the leading-order (LO) Alpgen 2.13 [36] generator interfaced to Herwig with the AUET2-CTEQ6L1 [30, 37] tune. The *W*/*Z*+jet simulated data include dedicated samples for heavy-flavour production ($b\bar{b}, c\bar{c}$ and *c*). Signal samples of $t\bar{t}\to H^{+}bW^{-}\bar{b}$ were generated using Pythia 6.425 for seven different *H* ^+^ masses from 90 GeV to 150 GeV.

The data are affected by the detector response to multiple *pp* interactions occurring in the same or neighbouring bunch crossings, known as pile-up. Minimum-bias interactions generated by Pythia 6.425 [34], which has been tuned to data [38], were overlaid on the simulated signal and background events. The events were weighted to reproduce the distribution of the number of interactions per bunch crossing observed in the data. A Geant4 simulation [39, 40] is used to model the response of the ATLAS detector, and the samples are reconstructed and analysed in the same way as the data.

## Physics objects and event selection

Jets are reconstructed from topological clusters of calorimeter cells [41] using the anti-*k*_*t*_ algorithm [42, 43] with a radius parameter *R*=0.4. Topological clusters are built using an algorithm that suppresses detector noise. Jets are corrected back to particle (truth) level using calibrations derived from Monte Carlo simulation and validated with both test-beam [44] and collision-data studies [45]. Events are excluded if they contain a high-*p*_T_ jet that fails quality criteria rejecting detector noise and non-collision backgrounds [46]. To suppress the use of jets originating from secondary *pp* interactions, a jet vertex fraction (JVF) algorithm is used. Inner detector tracks, with *p*_T_>1 GeV, are uniquely associated with jets using $\Delta R(\mathrm{jet, track}) < 0.4$, where $\Delta R\equiv\sqrt{( \Delta\phi)^{2} + ( \Delta\eta)^{2}}$. The JVF algorithm requires that at least 75 % of the sum of the *p*_T_ of the tracks associated with the jet is from tracks compatible with originating from the primary vertex of the event. Tagging algorithms identify jets originating from *b*-quark decays by selecting jets with tracks from secondary vertices or those with a large impact parameter significance. A multivariate algorithm (MV1) [47], which uses a neural network to combine the weights from multiple tagging algorithms, is used to identify jets originating from *b*-quarks. Jets passing the MV1 selection are referred to as *b*-tagged jets. The selection on the discriminating variable of the algorithm achieves an average per-jet efficiency of 70 % to select *b*-jets in $t\bar{t}$ events, with a probability to incorrectly tag light jets of less than 0.1 % [48]. Studies have shown that this working point has a 20–40 % efficiency to tag a *c*-jet, depending on the *p*_T_ of the jet [49].

Muons are required to be identified in both the ID and MS, and their momentum is obtained through a combined fit of all hits in both systems. Muons are also required to satisfy isolation criteria to reject those originating from heavy-flavour decays and hadrons misidentified as muons. The sum of the transverse momenta of ID tracks within a cone of Δ*R*=0.3 around the muon, excluding the muon track itself, is required to be less than 2.5 GeV. The transverse energy measured in the calorimeters within a cone of Δ*R*=0.2, excluding the energy associated with the muon, is required to be less than 4 GeV. In addition, muons are removed if they are found within Δ*R*<0.4 of a jet that has *p*_T_>25 GeV [50, 51].

The reconstruction of electron candidates starts from a seed cluster in the second layer of the EM calorimeter. The cluster is matched to a track found in the ID and a set of selection criteria are applied to reject electron candidates originating from jets [52]. Electrons are required to be isolated in order to suppress the QCD multi-jet background. The calorimeter isolation is performed using a cone of Δ*R*=0.2 and the track isolation uses a cone of radius Δ*R*=0.3. The calorimeter and track isolation cut values are chosen to achieve 90 % efficiency with respect to selected electron candidates [53]. As in the case of muons, the electron itself is excluded from the sum over the isolation cone.

Energy deposits in the calorimeter are expressed as four-vectors (*E*,**p**), where the direction is determined from the position of the calorimeter cluster and the nominal interaction point (*x*=*y*=*z*=0). The clusters are formed assuming *E*=|*p*|. The missing transverse momentum ($E_{\mathrm{T}}^{\mathrm{miss}}$) is given by the negative of the vector sum of the calorimeter four-momenta, projected into the (*x*,*y*) plane. The $E_{\mathrm{T}}^{\mathrm{miss}}$ calculation uses the energy scale appropriate for each physics object described above. For muons, the momentum measured from the combined tracking is used as the energy. The remaining calorimeter cells not associated with any physics object are included at the electromagnetic energy scale of the calorimeter [54].

A set of requirements is imposed to select events containing $t\bar{t}$ decays in the lepton+jets channel [50]. First, events are required to contain a primary vertex with at least five associated tracks to suppress non-collision backgrounds. Exactly one electron with a large transverse energy (*E*_T_>25 GeV) and |*η*|<2.5, excluding the barrel–endcap transition region 1.37<|*η*|<1.52, or one muon with large transverse momentum (*p*_T_>20 GeV) and |*η*|<2.5 is required. The selected lepton must match a lepton trigger object that caused the event to be recorded. Jets present in *W*/*Z*+jet events tend to originate from soft gluon emissions. These backgrounds are therefore reduced by requiring at least four jets with *p*_T_>25 GeV and |*η*|<2.5. At least two jets must be identified as originating from a *b*-decay using the MV1 algorithm. To suppress backgrounds from QCD multi-jet events, the missing transverse momentum is required to be $E_{\mathrm{T}}^{\mathrm {miss}}> 20 (30)~\mathrm{GeV}$ in the muon (electron) channel. Further reduction of the multi-jet background is achieved by requiring the transverse mass^2^ (*m*_T_) of the lepton and $E_{\mathrm{T}}^{\mathrm{miss}}$ to satisfy *m*_T_>30 GeV in the electron channel and $(E_{\mathrm{T}}^{\mathrm{miss}}+ m_{\mathrm{T}}) > 60~\mathrm {GeV}$ in the muon channel. These requirements favour the presence of a *W* boson, decaying to *ℓν*, in the final state. The selections are more stringent in the electron channel because of the larger multi-jet background.

## Kinematic fit

In the selected events, the two jets originating from the decay of the *H* ^+^ must be identified in order to reconstruct the mass. A kinematic fitter [17] is used to identify and reconstruct the mass of dijets from *W*/*H* ^+^ candidates, by fully reconstructing the $t\bar{t}$ system. In the kinematic fitter, the lepton, $E_{\mathrm {T}}^{\mathrm{miss}}$ (assumed to be from the neutrino), and four jets are assigned to the decay particles from the $t\bar{t}$ system. The longitudinal component of the neutrino momentum is calculated from the constraint that the invariant mass of the leptonic *W* boson decay products must be the experimental value (80.4 GeV) [55]. This leads to two possible solutions for this momentum. When complex solutions are returned, the real part of the solution is used in the fit. The fitter also constrains the invariant mass of the two systems (*bℓν*,*bjj*) to be within *Γ*_*t*_=1.5 GeV of the top-quark mass 172.5 GeV, which is consistent with the measured top-quark mass [56]. When assigning jets in the fitter, *b*-tagged jets are assumed to originate from the *b*-quarks. The best *bbjj* combination is found by minimising a *χ* ^2^ for each assignment of jets to quarks and for the choice of solution for the longitudinal neutrino momentum, where the five highest-*p*_T_ jets are considered as possible top-quark decay products. Since the *b*-jets are only allowed to be assigned to the *b*-quarks, and the two untagged jets are assigned to quarks from the same charged boson, there are two possible jet configurations overall for events with four jets, two of which are *b*-tagged. For events with at least five jets, the two highest-*p*_T_ jets are always assumed to be from the top-quark decay products (*W*/*H* ^+^ boson or *b*-quark) to reduce the combinatorics in the fit procedure. The combination with the smallest *χ* ^2^ value, $\chi^{2}_{\mathrm{min}}$, is selected as the best assignment. The function minimised in the fit is: 
1


In the first term, the fitted transverse momenta of the lepton and the four jets currently under consideration are allowed to vary around the measured values using the corresponding measured resolutions (*σ*_*i*_). In the fit only the magnitudes of the object *p*_T_s are varied; the angles of the jets and leptons are assumed to be measured with good precision. The vector sum of the momenta of the remaining jets (*p*_T_>15 GeV) in the event, labelled SEJ, is allowed to vary in the second term. The resolution for this term is taken from the nominal jet resolution. Letting the SEJ vary allows the $E_{\mathrm{T}}^{\mathrm{miss}}$ to be recalculated from the fitted values of its dominant components. Jets with lower *p*_T_ and energy from calorimeter cells not associated with any physics object are both minor contributions to the $E_{\mathrm{T}}^{\mathrm{miss}}$ and are held fixed in the re-calculation of the $E_{\mathrm{T}}^{\mathrm{miss}}$. The third term constrains the hadronic (*jjb*) and leptonic (*bℓν*) top-quark candidates to have a mass close to the top-quark mass.

**Fig. 1:**
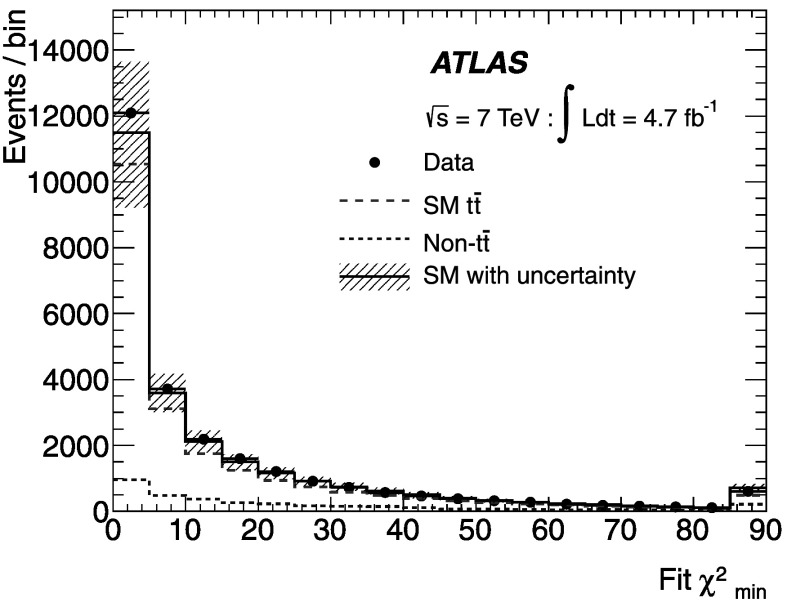
Comparison of the distribution of $\chi^{2}_{\mathrm{min}}$ from the kinematic fitter for data and the expectation from the background estimates for the combined electron and muon channels. The MC simulation is normalised to the expectation for the SM ($\mathcal{B} (t\to H^{+}b)=0$). The uncertainty shown on the background estimate is the combination in quadrature of the ±1*σ* systematic uncertainties. The final bin also contains the overflow entries

**Fig. 2:**
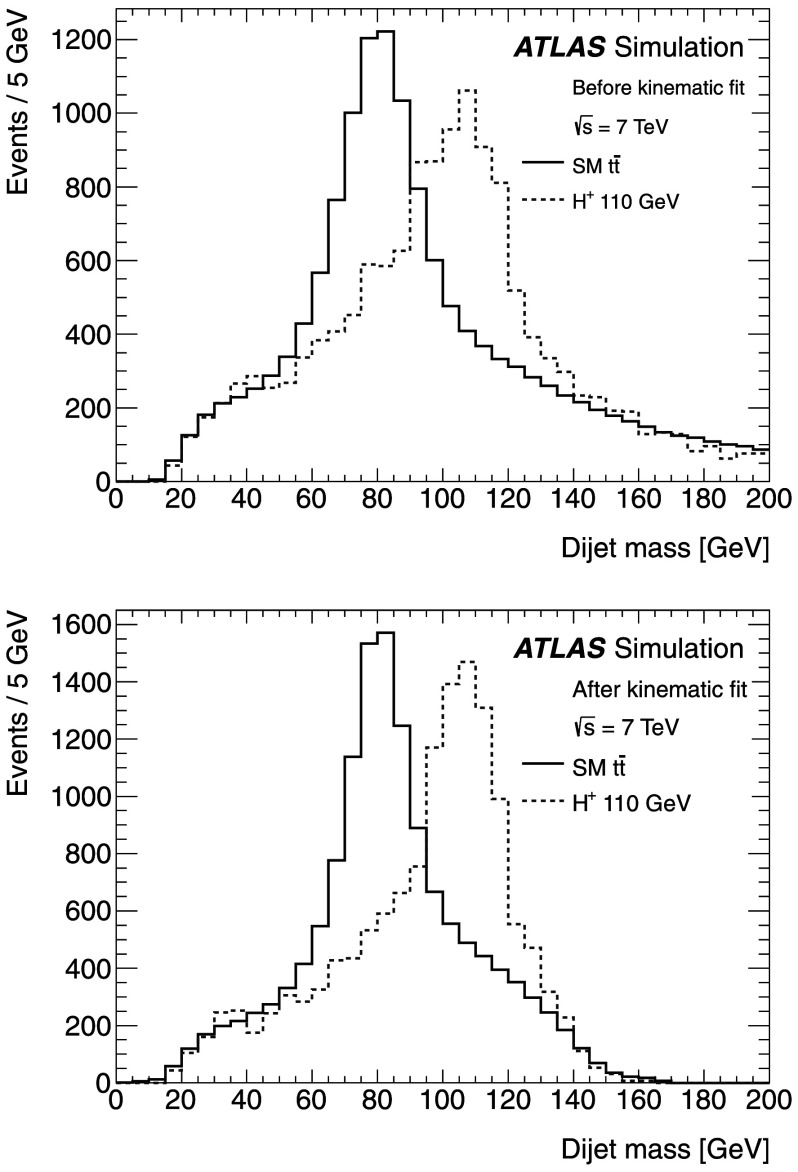
Comparison of the dijet mass distribution before (*upper part*) and after (*lower part*) the kinematic fit and the *χ* ^2^<10 selection criterion. The distribution is shown for MC simulations of SM $t\bar{t}$ decays and the $m_{H^{+}} = 110~\mathrm{GeV}$ signal ($t\bar{t} \rightarrow H^{+}bW^{-}\bar{b}$). The curves are normalised to the same area

The $\chi^{2}_{\mathrm{min}}$ distribution for selected events in the data agrees well with the expectation from the simulation (see Fig. 1). Events are required to have $\chi^{2}_{\mathrm{min}} < 10$ to remove poorly reconstructed $t\bar{t}$ events. This selection has an efficiency of 63 % for SM $t\bar{t}$ events. The fit results in a 12 GeV dijet mass resolution, as shown in Fig. 2. This is a 20–30 % improvement, depending on the mass of the boson studied, compared to the resolution obtained when the same jets are used with their original transverse momentum measurements. After the fit, there is better discrimination between the mass peaks of the *W* boson from SM decays of $t\bar{t}$ and a 110 GeV *H* ^+^ boson in this example.

Table 1 shows the number of events observed in the data and the number of events expected from the SM processes after the selection requirements. The SM $t\bar{t}$ entry includes events from both the lepton+jets and dilepton $t\bar{t}$ decay modes, where the dilepton events can pass the event selection if the events contain additional jets and the second lepton is not identified. Good agreement is observed between the data and the expectation. The table also shows the number of signal events expected for $\mathcal{B}(t \rightarrow H^{+}b)=10~\%$. The signal prediction accounts for acceptance differences due to the different kinematics of the *t*→*H* ^+^ *b* events relative to the SM *t*→*Wb* events.

**Table 1:** The expected numbers of events from SM processes, integrated over the full range of dijet masses and the observed number of events in the data after all the selection requirements. The expected number of events in the case of a signal with $m_{H^{+}}=110~\mathrm{GeV}$ and $\mathcal{B}(t\to H^{+}b)=10~\%$ is also shown. The $t\bar {t}\rightarrow W^{+}b W^{-}\bar{b}$ numbers include both the lepton+jets and dilepton decay channels. The uncertainties are the sum of the contributions from statistics and systematic uncertainties

Channel	Muon	Electron
Data	10107	5696
SM $t\bar{t}\rightarrow W^{+}b W^{-}\bar{b}$	8700±1800	5000±1000
*W*/*Z*+jets	420±120	180±50
Single top quark+Diboson	370±60	210±30
QCD multi-jet	300±150	130±60
Total expected (SM)	9800±1800	5500±1000
$m_{H^{+}} = 110~\mathrm{GeV}$		
$\mathcal{B}(t\to H^{+}b)=10~\%$:		
$t\bar{t}\rightarrow H^{+}bW^{-}\bar{b}$	1400±280	800±160
$t\bar{t}\rightarrow W^{+}b W^{-}\bar{b}$	7000±1400	4000±800
Total expected $(\mathcal{B}=10~\%)$	9500±1700	5300±1000

## Systematic uncertainties

The background estimates and the estimate of the signal efficiency are subject to a number of systematic uncertainties. The QCD multi-jet background is estimated using a data-driven method [57] that employs a likelihood fit to the $E_{\mathrm{T}}^{\mathrm{miss}}$ distribution in the data, using a template for the multi-jet background and templates from MC simulations for all other processes. The uncertainty on the QCD multi-jet background is evaluated to be 50 % by studying the effect of pile-up events on the fit results and by performing likelihood fits on the *m*_T_(*W*) distribution. The dijet mass distribution of multi-jet events is obtained from a control region in the data, where leptons are required to be semi-isolated, such that the transverse momentum of the inner detector tracks in a cone of radius Δ*R*=0.3, excluding the lepton, satisfies $0.1<p_{\mathrm{T}}^{\Delta R = 0.3}/p_{\mathrm{T}}(e,\mu)<0.3$. Leptons in the control region are also required to have a large impact parameter with respect to the identified primary vertex (0.2 mm<|*d*_0_|<2 mm) and an impact parameter significance $|d_{0}|/\sigma_{d_{0}}>3$.

The rate of *W*+jets events is estimated by a data-driven method [58] that uses the observed difference in the number of *W* ^+^ and *W* ^−^ bosons in the data and the charge asymmetry (*W* ^+^−*W* ^−^)/(*W* ^+^+*W* ^−^), which is calculated to good precision by the MC simulation of *W*+jets events. The heavy flavour fraction of the *W*+jets MC simulation is calibrated using *W*+1 jet or *W*+2 jets events in the data. The uncertainty on the *W*+jets background is 26 % (28 %) for the electron (muon) channel, which includes the uncertainty from the charge asymmetry and heavy flavour fraction components. The shape of the *m*_*jj*_ distribution for *W*+jets events is obtained from simulation.

Uncertainties on the modelling of the detector and on theory give rise to systematic uncertainties on the signal and background rate estimates. The following systematic uncertainties are considered: integrated luminosity (3.9 %) [22, 23], trigger efficiency (3.5 %/1 % for electron/muon), jet energy scale (1–4.6 %) [45], jet energy resolution (up to 16 % smearing) [59], and *b*-jet identification efficiency (5–17 %). The last three uncertainties depend on the *p*_T_ and *η* of the jets. Uncertainties on lepton reconstruction and identification efficiency are determined using a tag and probe method in samples of *Z* boson and *J*/*ψ* decays [60]. The momentum resolution and scales are determined from fits to samples of *W* boson, *Z* boson, and *J*/*ψ* decays [53, 61]. Additional *p*_T_-dependent uncertainties are placed on the *b*-jet (up to 2.5 %) and *c*-jet (up to 1.3 %) energy scales [45]. Uncertainties on the modelling of the $t\bar{t}$ background are estimated using a second MC generator (Powheg [62–64]) and comparing the effect of using Pythia and Herwig to perform the parton showering and hadronisation. Uncertainties on initial and final state radiation (ISR/FSR) are assessed using AcerMC interfaced to Pythia and examining the effects of changing the ISR/FSR parameters in a range consistent with experimental data [65]. The predicted SM $t\bar{t}$ cross-section for *pp* collisions at $\sqrt {s} = 7~\mathrm{TeV}$, obtained from approximate next to next to LO QCD calculations, is $\sigma_{t\bar{t}} = 167^{+17}_{-18}~\mathrm{pb}$ for a top-quark mass of 172.5 GeV [66]. The uncertainty on the predicted value includes the uncertainty in the renormalisation and factorisation scales, parton density functions, and the strong coupling constant. An additional uncertainty on the $t\bar{t}$ cross-section (4.5 %) is included due to the uncertainty on the top-quark mass. The uncertainty on the top-quark mass is 0.9 GeV from the combined measurement [56] at the Tevatron. However, this result would be biased in the presence of a $H^{+}\rightarrow c\bar{s}$ signal in the lepton+jets channel, so a larger uncertainty of 1.5 GeV is taken, which is consistent with the latest top-quark mass measurement in the dilepton channel from the CMS experiment [67]. Changing the top-quark mass leads to altered event kinematics, which results in a final uncertainty on the event rate of 1.9 %. The effects of these systematic uncertainties on the overall normalisation are listed in Table 2. The jet energy calibration, *b*-jet identification, $t\bar{t}$ background modelling, and ISR/FSR uncertainties also modify the shape of the dijet mass distribution and are therefore determined as a function of *m*_*jj*_. The systematic uncertainties that affect the shape of the *m*_*jj*_ distribution (top half of Table 2) are more important than the shape-independent uncertainties. The effects of the systematic uncertainties are comparable, within 10 % , between the SM and signal $t\bar{t}$ samples. The combined uncertainty on the single top-quark and diboson backgrounds is 15 %, which comes mostly from the uncertainties on the cross-section, jet energy scale, and *b*-tagging. The total uncertainty on the overall normalisation of the non-$t\bar{t}$ backgrounds is 30 %.

**Table 2:** Effect of the systematic uncertainties on the event rate of $t\bar{t}$ background and signal ($m_{H^{+}}=110~\mathrm{GeV}$) events before any reduction from the likelihood fit, described in Sect. 6

Systematic source	
Shape dependent	
Jet energy scale	±9.5 %
*b*-jet energy scale	+0.3,−0.6 %
*c*-jet energy scale	+0.1,−0.3 %
Jet energy resolution	±0.9 %
MC generator	±4.3 %
Parton shower	±3.1 %
ISR/FSR	±8.8 %
Shape independent	
*b*-tagging efficiency (b-jets)	±11 %
*b*-tagging efficiency (c-jets)	±2.4 %
*b* mistag rate	±1.8 %
Lepton identification	±1.4 %
Lepton reconstruction	±1.0 %
*t*-quark mass	±1.9 %
$t\bar{t}$ cross-section	+10,−11 %
Luminosity	±3.9 %

## Results

**Fig. 3:**
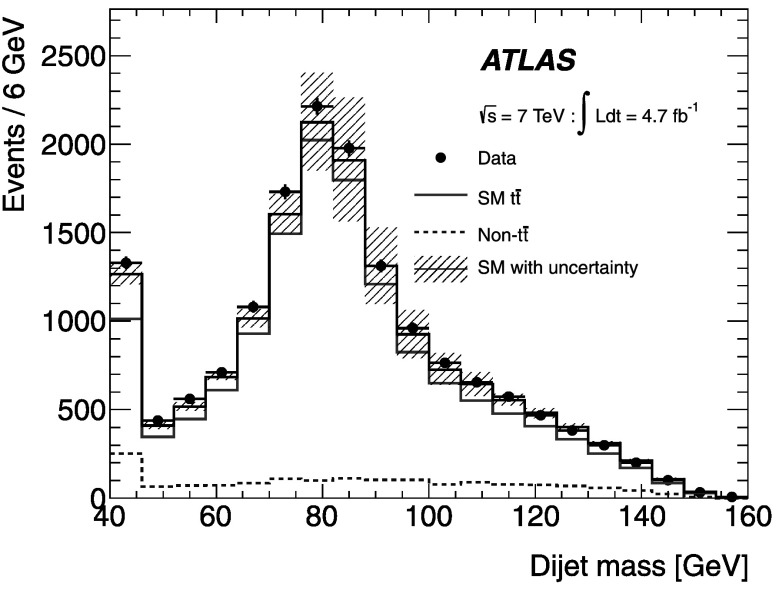
The dijet mass distribution from data and the expectation from the SM ($\mathcal{B}=0$). The *error bars* represent the statistical uncertainty on the data. The uncertainty shown on the background estimate is the combination in quadrature of the ±1*σ* systematic uncertainties, accounting for the constraint from the profile likelihood fit. The first and last bins contain the underflow and overflow events respectively

The data are found to be in good agreement with the distribution of the dijet mass expected from SM processes (see Fig. 3). The fractional uncertainty on the signal-plus-background model is comparable to the background only model. Upper limits on the branching ratio $\mathcal{B}(t\rightarrow H^{+}b)$ are extracted as a function of the charged Higgs boson mass. The upper limits are calculated assuming the charged Higgs always decays to $c\bar{s}$. The following likelihood function is used to describe the expected number of events as a function of the branching ratio:

2 $$ \mathcal{L}(\mathcal{B}, {\mathbf{\alpha}}) = \prod _i \frac{\nu _{i}(\mathcal{B} ,\alpha )^{n_i} e^{-\nu_{i}(\mathcal{B},\alpha)}}{n_i!} \prod_j \frac {1}{\sqrt {2\pi}} e^{-\frac{\alpha_j^2}{2}}, $$

where *n*_*i*_ is the number of events observed in bin *i* of the dijet mass distribution and *j* labels the sources of systematic uncertainty. The number of expected signal plus background events in each bin, $\nu_{i}(\mathcal{B},\alpha)$, is given by 
3 where $n^{N}_{i}$ is the expected number of non-$t\bar{t}$ background events, $\sigma_{t\bar{t}}$ is the cross-section for $t\bar{t}$ production, $\mathcal{L}$ is the integrated luminosity, $\mathcal{B}$ is the branching ratio of *t*→*H* ^+^ *b*, and $A^{H^{+}}$ and *A* ^*W*^ are the acceptances for signal ($t\bar {t}\to H^{+}b\ell\nu\bar{b}$) and SM $t\bar{t}$ ($t\bar{t}\to jjb\ell\nu \bar {b}$ and $t\bar{t}\to\ell\bar{\nu} b\ell\nu\bar{b}$) events respectively. The decay mode $t\bar{t}\rightarrow H^{+}bH^{-}\bar{b}$ does not contribute to the expectation because this mode does not produce a single isolated lepton and hence has a negligible efficiency to pass the selection requirements. The $S_{i}^{H^{+}}$ ($S_{i}^{W}$) parameter describes the shape of the *m*_*jj*_ spectrum (normalised to one) for *H* ^+^ (*W*) boson production. It gives the relative number of events in bin *i* according to the normalised *m*_*jj*_ distribution. The *α*_*j*_ variables are nuisance parameters representing the systematic uncertainties, which are constrained via the Gaussian terms in Eq. (2). The effect of the systematic uncertainties on the non-$t\bar{t}$ background can be obtained by calculating the effect of each source of uncertainty on each non-$t\bar{t}$ background component, and combining them in quadrature. Since this sum is dominated by the uncertainties on the data-driven *W*+jets and multi-jet background estimates, the combined variation is treated as a single nuisance parameter (*α*_*b*_, *b*∈*j*) and is assumed to be uncorrelated from the other systematic uncertainties. The *ρ*_*ji*_ functions account for the effect of nuisance parameters on the yields and are defined such that *ρ*_*ji*_(*α*_*j*_=±1*σ*) represents the 1±1*σ* fractional change in the number of entries in bin *i* of the dijet mass spectrum due to systematic uncertainty *j*. The physics measurement involves a sufficiently large number of events that this likelihood can constrain the *α*_*j*_ parameters beyond the precision of the subsidiary measurements. The effects of systematic uncertainties are applied coherently in signal and background distributions. The subsidiary measurements of the *α*_*j*_ parameters are taken to be uncorrelated. The fit uses 17 nuisance parameters in total. None of them are shifted by more than one sigma compared to the original values obtained in subsidiary measurements. Maximal reduction of uncertainty is obtained for the jet energy scale parameter which is reduced by 50 %.

**Fig. 4:**
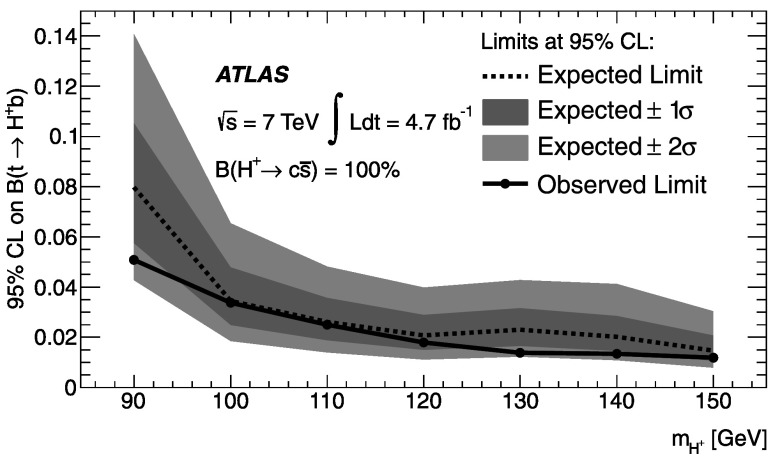
The extracted 95 % CL upper limits on ${\mathcal{B}}(t \rightarrow H^{+}b)$, assuming that $\mathcal{B}(H^{+}\rightarrow c\bar{s}) = 100~\% $, are shown for a range of charged Higgs masses from 90 GeV to 150 GeV. The limits shown are calculated using the CL_s_ limit-setting procedure

The limits on the branching ratio are extracted using the CL_s_ technique at 95 % confidence level [68, 69]. The consistency of the data with the background model can be determined by comparing the value of the test statistic (a profile likelihood ratio based on Eq. (2)) in the data with the expectation from background-only Monte Carlo simulated experiments. The corresponding probability (*p*-value) for the background to produce the observed mass distribution varies from 67 % to 71 % as a function of $m_{H^{+}}$, indicating that there is no significant deviation from the background hypothesis. The expected and observed limits, shown in Table 3 and Fig. 4, are calculated using asymptotic formulae [68]. The expected limits on $\mathcal{B}$, including both statistical and systematic uncertainties, vary between 1–8 % depending on $m_{H^{+}}$; if only the statistical uncertainty is considered these limits are 1–3 %. The observed limits, including both statistical and systematic uncertainties, vary between 1–5 %. The extracted limits are the most stringent to date on the branching ratio $\mathcal{B}(t\to H^{+}b)$, assuming $\mathcal{B}(H^{+}\to c\bar{s})=100~\%$. These results can be used to set limits for a generic scalar charged boson decaying to dijets in top-quark decays, as long as the width of the resonance formed is less than the experimental dijet resolution of 12 GeV.

**Table 3:** Expected and observed 95 % CL limits, including systematic uncertainties, on the branching ratio for a top-quark to decay to a charged Higgs boson and a *b*-quark, assuming that $\mathcal{B}(H^{+}\rightarrow c\bar {s}) = 100~\%$. The limits shown are calculated using the CL_s_ limit-setting procedure

Higgs mass	Expected limit (stat.⊕ syst.)	Observed limit (stat.⊕ syst.)
90 GeV	0.080	0.051
100 GeV	0.034	0.034
110 GeV	0.026	0.025
120 GeV	0.021	0.018
130 GeV	0.023	0.014
140 GeV	0.020	0.013
150 GeV	0.015	0.012

## Conclusions

A search for charged Higgs bosons decaying to $c\bar{s}$ in $t\bar{t}$ production has been presented. The dijet mass distribution is in good agreement with the expectation from the SM and limits are set on the branching ratio $\mathcal{B}(t\to H^{+}b)$, assuming $\mathcal{B}(H^{+}\to c\bar{s})=100~\%$. The observed limits range from $\mathcal{B}=5~\%$ to 1 % for $m_{H^{+}}=90$ GeV to 150 GeV. These are the best limits to date on charged Higgs boson production in this channel.

## Footnotes

1 ATLAS uses a right-handed coordinate system with its origin at the nominal interaction point (IP) in the centre of the detector and the *z*-axis along the beam pipe. The *x*-axis points from the IP to the centre of the LHC ring, and the *y*-axis points upward. Cylindrical coordinates (*r*,*ϕ*) are used in the transverse (*x*, *y*) plane, *ϕ* being the azimuthal angle around the beam pipe. The pseudorapidity is defined in terms of the polar angle *θ* as *η*=−lntan(*θ*/2).

2
$m_{\mathrm{T}}=\sqrt{2p_{\mathrm {T}}^{\ell}E_{\mathrm{T}}^{\mathrm{miss}}(1-\cos\Delta\phi)}$ where Δ*ϕ* is the azimuthal angle between the lepton and the missing transverse momentum.
